# Trace-Level Ammonia–Water Interactions in Hydrogen: Challenges in Gas Purity Analysis Using Optical-Feedback Cavity-Enhanced Absorption Spectroscopy (OF-CEAS)

**DOI:** 10.1021/acsmeasuresciau.5c00105

**Published:** 2025-10-13

**Authors:** Mehmet Emin Bayat, Heinrich Kipphardt, Carlo Tiebe, Dirk Tuma, Carsten Engelhard

**Affiliations:** † 42220 Bundesanstalt für Materialforschung und -prüfung (BAM), Richard-Willstätter-Str. 11, 12200 Berlin, Germany; ‡ Department of Chemistry and Biology, and Center of Micro- and Nanochemistry and (Bio-)Technology (Cμ), 14312 University of Siegen, Adolf-Reichwein-Str. 2, 57068 Siegen, Germany

**Keywords:** hydrogen, ammonia, humidity, OF-CEAS, adsorption, surface interactions

## Abstract

Ammonia is a critical impurity in hydrogen fuel due to its irreversible poisoning effect on proton exchange membrane fuel cells. Therefore, international standards (e.g., ISO 14687) set a stringent threshold of 100 nmol/mol. Furthermore, with the growing potential use of ammonia as a hydrogen carrier, its accurate quantification is becoming increasingly important. However, the presence of trace humidity poses analytical challenges, as ammonia may interact with water or interfaces, thereby affecting its detectability. Therefore, the goal of this work is to enable accurate trace ammonia quantification for hydrogen purity measurements through fundamental studies of the methodological challenges. Here, low-pressure sampling (ultra)­long-path Optical-Feedback Cavity-Enhanced Absorption Spectroscopy (OF-CEAS) was applied with an effective optical path length of approximately 6.17 km. We studied three average amounts of ammonia: (38.2 ± 0.8) nmol/mol, (74.8 ± 0.7) nmol/mol, and (112.1 ± 1.2) nmol/mol. Furthermore, these amounts were investigated at trace-humidity levels ranging from 0.8 to 8.5 ppm_V_. We observed a systematic, nonlinear, and humidity-dependent positive measurement bias of up to + (1.0 ± 0.2) nmol/mol at the maximum investigated trace-humidity volume fraction of 8.5 ppm_V_. This bias was not caused by spectral interference but rather by water-induced accumulation of ammonia within the optical cavity. Moreover, time-resolved measurements in the presence of trace ammonia showed that water desorption follows first-order kinetics, whereas water adsorption followed mixed-order kinetics with an apparent reaction order of 1.57 ± 0.03. Distinct hydration states of surface-bound ammonia were identified, whereas under dry conditions and with increasing amounts of ammonia, enhanced surface adhesion through intermolecular clustering was observed. In addition, the presence of ammonium species within the sorption layer was indirectly confirmed by our experiments. In conclusion, we provide a deeper insight into trace-level ammonia–water interactions and establish a framework for optimizing methodologies, particularly for (ultra)­long-path optical gas measurement systems.

## Graphical abstract

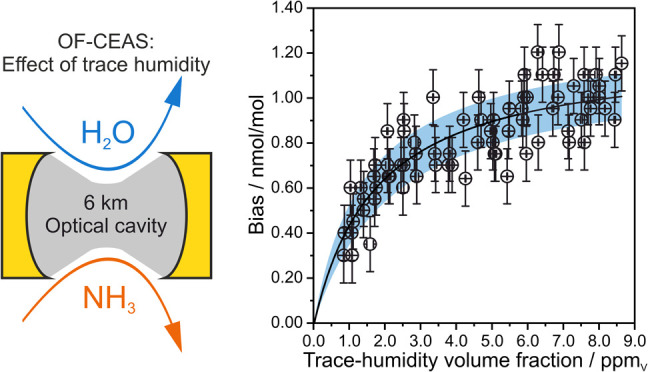

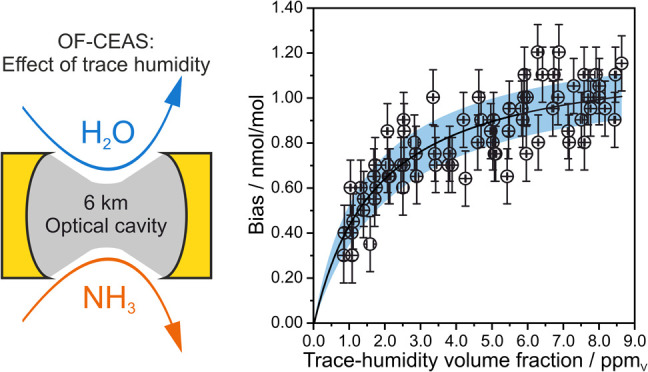

## Introduction

Ammonia is used as refrigerant^1,2^ and in the production of fertilizers,^3,4^ plastics,^4,5^ explosives,^4,6,7^ and synthetic fibers.^4,8^ Around 175 million tons are produced worldwide each year using the Haber-Bosch process, which corresponds to a market valuation of around USD 70 billion.^9^ The Haber-Bosch process is employed to synthesize ammonia by reacting hydrogen with nitrogen.^10,11^ Traditionally, hydrogen is derived from steam reforming of coal or natural gas (black or gray hydrogen). This fossil-based route accounts for the majority of the process’s CO_2_ emissions but could be replaced with green hydrogen produced via electrolysis.^12–14^ This modification alone would reduce the average well-to-gate CO_2_e emission footprint of Haber-Bosch process-based ammonia plants by approximately 85%.^15^ However, at the same time, new approaches for a sustainable ammonia production are also being investigated, such as the use of the earth’s subsurface as a natural reactor for geo-ammonia production.^16^

Beyond its conventional applications, ammonia is increasingly recognized as a resource for the energy transition.^9,17–19^ It can be used directly as an energy fuel^20–22^ and is regarded as an energy transport medium.^9,19,23,24^ Unlike hydrogen, ammonia can be liquefied very easily, making it significantly simpler to store and ship while binding 1.5 molar quantities of hydrogen in liquid form. At its final destination, it can be catalytically split back into hydrogen and nitrogen,^25–27^ thus allowing the recovered hydrogen to be used in various applications, such as the production of green steel by direct reduction,^28–30^ as fuel for heavy-duty vehicles powered by proton exchange membrane (PEM) fuel cells,^31–33^ to supply the gas grid with hydrogen-enriched natural gas^34–36^ or for a dedicated hydrogen-based gas grid.^37–39^

For applications utilizing hydrogen as a fuel, PEM fuel cells are the preferred technology for power conversion due to their high efficiency and environmental benefits. In this process, hydrogen undergoes an electrochemical reaction with oxygen to generate electricity and water as the primary by-product.^40^ Modern fuel cells achieve an efficiency of approximately 60%,^31^ significantly outperforming conventional hydrogen combustion, which only reaches approximately 40%.^41^ However, hydrogen used in fuel cells must meet rigorous purity requirements, classified as grade D according to ISO 14687.^42^ This standard describes a hydrogen purity of at least 99.97%, with strict threshold limits for specific contaminants. Such high purity is required because of the fuel cell’s sensitivity to impurities, which can affect their long-term stability and operational lifespan.^43^ Some contaminants temporarily degrade performance in a reversible manner, while others cause irreversible damage. A particularly critical impurity is ammonia, as it irreversibly degrades fuel cell performance – for example, by reducing the membrane’s ionic conductivity through the replacement of protons (H^+^) with ammonium ions (NH_4_^+^).^44,45^ To mitigate this risk, ISO 14687 has established an ammonia threshold of 100 nmol/mol.^42^ Given the growing perspectives of ammonia as a hydrogen carrier in the supply chain, the importance of precise quantification of ammonia impurities after the conversion process is both evident and essential.

Ensuring reliable and validated hydrogen purity measurements at these levels represents a significant analytical challenge. While several methods have been developed for hydrogen purity assessment,^43,46,47^ one has demonstrated particular promise due to its high sensitivity, rapid response time, and user-friendly handling: low-pressure sampling Optical-Feedback Cavity-Enhanced Absorption Spectroscopy (OF-CEAS). This technique has proven highly effective across a wide range of use cases,^48–50^ with particularly strong performance in trace gas analysis.^51–54^ Therefore, in this study, we employed (ultra)­long-path OF-CEAS, with an effective optical path length of approximately 6.17 km, specifically for the quantification of trace levels of ammonia in hydrogen.

In addition, according to ISO 14687, grade D hydrogen may contain up to 5 μmol/mol of humidity, an amount that is substantially higher than the rigorous limit of just 100 nmol/mol ammonia.^42^ This significant disparity raises an important question: can ammonia still be reliably quantified at such low quantities, or does it interact with water and adhere to surfaces/interfaces within the piping, sampling system, or instrumentation? Considering these uncertainties, the central focus of our investigation was to assess whether (ultra)­long-path OF-CEAS is susceptible to interference from water when measuring ammonia at trace amounts.

To address these questions, we applied a dynamic dilution setup of our gravimetrically prepared gas standards to identify and quantify nonlinear, systematic, and trace-humidity-induced biases in OF-CEAS measurements. These biases, which emerged from an unexpected effect, were characterized across a range of trace-humidity volume fractions from 0.8 to 8.5 ppm_V_. In addition, we examined surface-mediated phenomena and described how water interacts with ammonia at interfaces and whether these effects are reversible or irreversible. Moreover, by determining reaction kinetics and discussing reactivities, we provide deeper insight into the interactions between ammonia and water in the trace-amount range, thereby building a framework for optimizing methodologies for the quantification of trace ammonia.

## Materials and Methods

### Reference Materials

A binary ammonia gas standard was used in this study, containing (370.08 ± 0.22) μmol/mol of ammonia in hydrogen, which was gravimetrically prepared in-house in accordance with ISO 6142–1.^55^ Therefore, (1.018 ± 0.001) g of ammonia (grade 6.0, Linde GmbH), sampled from the liquid phase and introduced by evaporation via a 25 mL cylinder, was diluted with (120.555 ± 0.015) g of hydrogen (grade 6.6, Air Products) in an aluminum alloy L6X gas cylinder (10 dm^3^) purchased from Luxfer Gas Cylinders. This yielded (998.73 ± 0.49) μmol/mol of ammonia in hydrogen, which served as a premixture (cylinder number 3096–231025). Subsequently, (44.882 ± 0.015) g of the premixture 3096–231025 was further diluted with (75.676 ± 0.015) g of hydrogen, also in an L6X gas cylinder, to create the final gas standard of (370.08 ± 0.22) μmol/mol ammonia in hydrogen (cylinder number 4114–240528). This cylinder served as certified reference material (CRM 1) for further dynamic dilution.

For carbon dioxide-containing experiments, the primary gas standard used was also prepared in-house in accordance with ISO 6142–1 ^55^ by mixing (89.625 ± 0.015) g of hydrogen with (19.584 ± 0.001) g of carbon dioxide (grade 5.5, Air Liquide), also introduced by evaporation via a 75 mL cylinder, into an L6X gas cylinder, yielding (9911.28 ± 0.59) μmol/mol carbon dioxide in hydrogen (cylinder number 4060–241111). This cylinder served as CRM 2 and was used to probe the potential surface reactivity of adsorbed ammonia species.

### Experimental Setup

Figure 1 shows a schematic illustration of the experimental setup. Sample gases containing trace ammonia in trace-humidified hydrogen were prepared by applying dynamic dilution using thermal bypass mass flow controllers (MFC) of the EL-FLOW Prestige series from Bronkhorst Deutschland Nord GmbH. Hydrogen (grade 5.0, Linde GmbH) was passed through a GateKeeper GPU gas purifier from Entegris Inc. (Model MC190–503) generating a trace-humidity volume-fraction baseline consistently below 0.5 ppm_V_. This served as a clean and dry hydrogen supply for MFC 1 and MFC 2, with an upstream operating pressure of 3 bar­(a).

**Figure 1:**
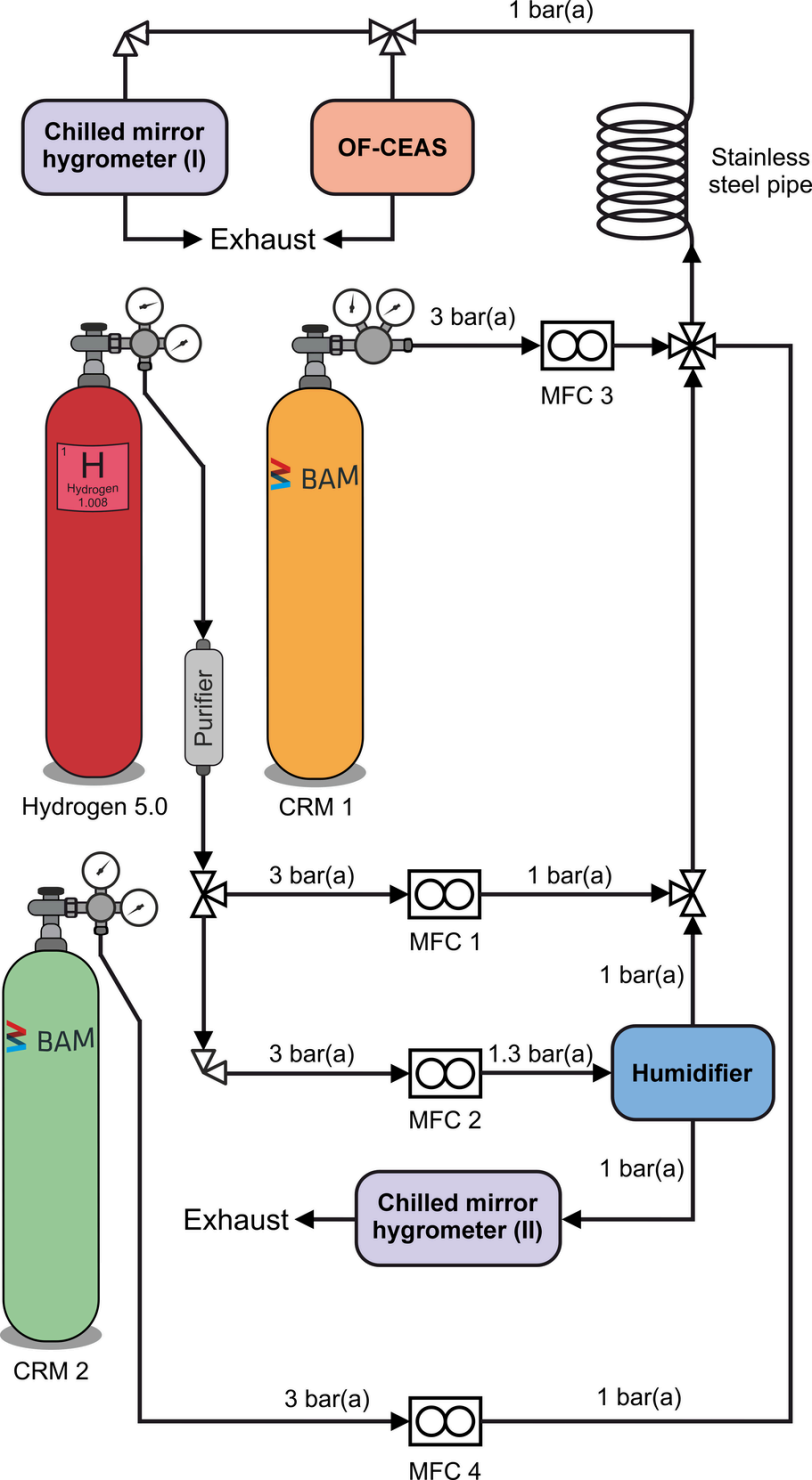
Schematic representation of the experimental setup. An ammonia-containing CRM was dynamically diluted with purified hydrogen to generate defined amounts of ammonia in hydrogen. Additionally, humidification was performed using a humidification unit based on the saturation method to introduce humid hydrogen, enabling the preparation of trace-humidified gas mixtures. For the carbon dioxide-containing experiments, CRM 2 was introduced as third dry partial gas flow.

CRM 1 was supplied to MFC 3 at an upstream operating pressure of 3 bar­(a) to generate a partial gas flow containing ammonia. Three average trace amounts of ammonia were dynamically generated and investigated in this study: (i) (38.2 ± 0.8) nmol/mol, (ii) (74.8 ± 0.7) nmol/mol, and (iii) (112.1 ± 1.2) nmol/mol of ammonia in hydrogen. For this purpose, MFC 3 generated partial gas flow rates of 0.14, 0.28, and 0.40 mL_n_/min, respectively, which were subsequently diluted with a constant hold flow rate of 1500 mL_n_/min of hydrogen from MFC 1. The combined partial gas flows of MFC 1 and MFC 3 served as dry partial gas flow.

In this study, trace-humidity volume fractions are expressed in units of ppm_V_ or ppb_V_, rather than mole fractions, such as μmol/mol or nmol/mol. Doing so is based on the measurement principle of the chilled mirror hygrometer, which determines the partial pressure of water vapor – an absolute thermodynamic property. The selected units thus provide a direct correspondence to measured quantities.

For trace humidification, we utilized our in-house-developed humidification unit (HU), which is described in the Supporting Information (SUI). The HU is supplied by MFC 2 at a constant hold flow rate of 135 mL_n_/min of dry hydrogen. Humidification was performed via the saturation method^56^ with deionized water and at a constant operating pressure of (1.300 ± 0.002) bar­(a) and a saturation temperature of (15.2 ± 0.2) °C, resulting in an average saturation-humidity volume fraction of (13743 ± 15) ppm_V_. The HU is equipped with low-Δ*p*-MFCs from Bronkhorst, enabling the controlled generation of a humidified partial gas flow into the dry partial gas flow. In this study, humidified flow rates ranging from 0.25 mL_n_/min to 1.20 mL_n_/min were used to generate controlled trace-humidity volume fractions in the sample gases between 0.8 ppm_V_ and 8.5 ppm_V_. The excess humidified hydrogen was directed into an MBW 473-SHX reference chilled mirror hygrometer (SN 20–0330, MBW Calibration AG) with a valid calibration according to ISO 17025 ^57^ (Cert. No.: 7939MBW2020). This setup allows real-time, in situ quantification of the saturation at any measurement point.

For the carbon dioxide-containing experiments, CRM 2 was connected to MFC 4, with an upstream operation pressure of 3 bar­(a), to generate a flow rate of 3.25 mL_n_/min as a third dry partial gas flow. The dynamic dilution of CRM 2 into the dynamically prepared ammonia sample gases resulted in a carbon dioxide amount of 21.00 μmol/mol.

All dynamically mixed gases were fed into the measuring equipment through a 1/8″ Swagelok stainless-steel pipe (material number: 1.4571) with a length of 3 m. The length of the pipe was intentionally chosen because we suspected that surface interactions would be amplified by increasing the available surface area at such low amounts of ammonia. While no dedicated surface characterization was performed, the stainless-steel pipe used is of standard, non-roughened process quality.

### Measurements

Low-pressure sampling OF-CEAS was performed to quantify the amounts of ammonia and carbon dioxide in the hydrogen matrix. The applied ProCeas spectrometer was built by AP2E SAS (DURAG Holding AG). The system operated with an effective optical path length (OPL) of approximately 6.17 km, corresponding to a photon lifetime of 20.57 μs, for the quantification of ammonia. For carbon dioxide quantification, the effective OPL was approximately 3.54 km, with a corresponding photon lifetime of 11.80 μs. In both cases, the gas cells were operated at a pressure of 100 mbar­(a) and maintained at a temperature of 45 °C. The free optical beam path was purged with 500 mL_n_/min of nitrogen (grade 5.0, Linde GmbH). AP2E’s implemented factory calibration was used for ammonia quantification. Currently, the accuracy of the ammonia factory calibration cannot be fully verified, as establishing traceability to the SI base unit kilogram at such low amounts of substance presents significant challenges – particularly due to the reactivity and stickiness of ammonia. Nonetheless, preliminary results indicate that the ammonia readings are overestimated by approximately 12% (within the investigated amounts of ammonia), although a full assessment has not yet been completed. Carbon dioxide quantification was traced back to BAM standards with an accuracy of <0.1%. The measurement range for ammonia was between 1 nmol/mol and 10 μmol/mol, whereas the measurement range for carbon dioxide was between 50 nmol/mol and 50 μmol/mol. For quantitative results, AP2E utilizes a constrained fitting procedure with reduced degrees of freedom to accurately determine the area under the absorption peak. This approach operates with simultaneous fitting of the baseline, ensuring a robust and precise peak area estimation. Further, the OF-CEAS continuously sampled approximately 250 mL_n_/min of the sample gas through the low-pressure sampling, utilizing a restrictor with a nozzle diameter of 100 μm. Readings of the OF-CEAS were generated every 10 s.

The laboratory temperature was strictly controlled to ensure accurate ammonia quantification, as the measurements were highly sensitive to ambient temperature fluctuations. To stabilize conditions, both a central and an additional mobile air conditioning system were used. The laboratory was also operated under low-light conditions and shielded from sunlight. Even minor increases in ambient temperature of more than 0.5 °C (e.g., caused by lighting or sunrise) significantly increased ammonia readings due to temperature-induced desorption processes in the piping. This effect is particularly pronounced due to the high sensitivity and rapid response (<2 s) of the OF-CEAS system. Furthermore, the limit of detection (LOD) for ammonia is 1.4 nmol/mol (3.3 sigma).

The manufacturer of the OF-CEAS system did not disclose the wavelength for ammonia quantification, as it is considered proprietary information. Therefore, the emission of the ammonia laser mounted within the OF-CEAS was measured using an OSA305-Redstone Fourier Transform Optical Spectrum Analyzer (1.9 GHz resolution, 1.0–5.6 μm, Thorlabs Inc.). For this purpose, the free optical beam path was coupled into an optical fiber (Thorlabs Inc., M28L01 SMA-SMA) and analyzed. While the emission wavelength was determined with an accuracy of 0.45 nm, it is reported here only approximately as (2.2 ± 0.2) μm to preserve the confidentiality of the proprietary information.

Trace-humidity volume fractions of the ammonia containing sample gases were quantified using the MBW 373-LX reference chilled mirror hygrometer (SN 19-0416, MBW Calibration AG) by measuring the frost point temperatures. The hygrometer was calibrated according to ISO 17025 ^57^ (Cert. No.: 7122MBW2019). Readings of the chilled mirror hygrometer were generated every 10 s using the Software Gecko R2 (Version: 0.9.7.2326, MBW Calibration AG). The flow rate through the hygrometer was approximately 1250 mL_n_/min.

Pressure measurements in the piping system were performed using pressure transmitters from KELLER Druckmesstechnik AG (Model PAA-23SX), with one transmitter located in the HU and another positioned in the piping system after mixing the dry and humid partial gas flows. Additionally, both chilled mirror hygrometers feature an internal pressure sensor on the sensor head.

Laboratory temperature measurements were conducted using an external PT100 Class A temperature sensor from the MBW 373-LX, which was mounted on a stainless-steel pipe.

## Results and Discussion

### Time-Dependent Surface Dynamics

To investigate ammonia–water surface interactions and potential measurement biases of the OF-CEAS, a standardized procedure with a series of measurements was applied. As mentioned earlier, three average amounts of ammonia – (38.2 ± 0.8) nmol/mol, (74.8 ± 0.7) nmol/mol, and (112.1 ± 1.2) nmol/mol – were investigated in trace-humidified hydrogen ranging from (0.8–8.5) ppm_V_, with humidity being introduced from a thermodynamically equilibrated dry state. Thermodynamic equilibrium was assumed once the ammonia signal remained stable within ± 0.5 nmol/mol for 2 h, whereas a dry state was defined by trace-humidity volume fractions below < 0.5 ppm_V_.

As exemplarily illustrated in the time series shown in Figure 2a, each measurement cycle consisted of a 3-h dry prephase, a 6-h humidification phase, and a 3-h dry postphase, resulting in a total duration of 12 h per individual measurement point. These extended periods ensured transitions between thermodynamically stable states. However, the entire experimental measurement campaign comprised 79 measurement cycles (see SUI Table S1) that were analyzed. The results are presented and discussed below.

**Figure 2:**
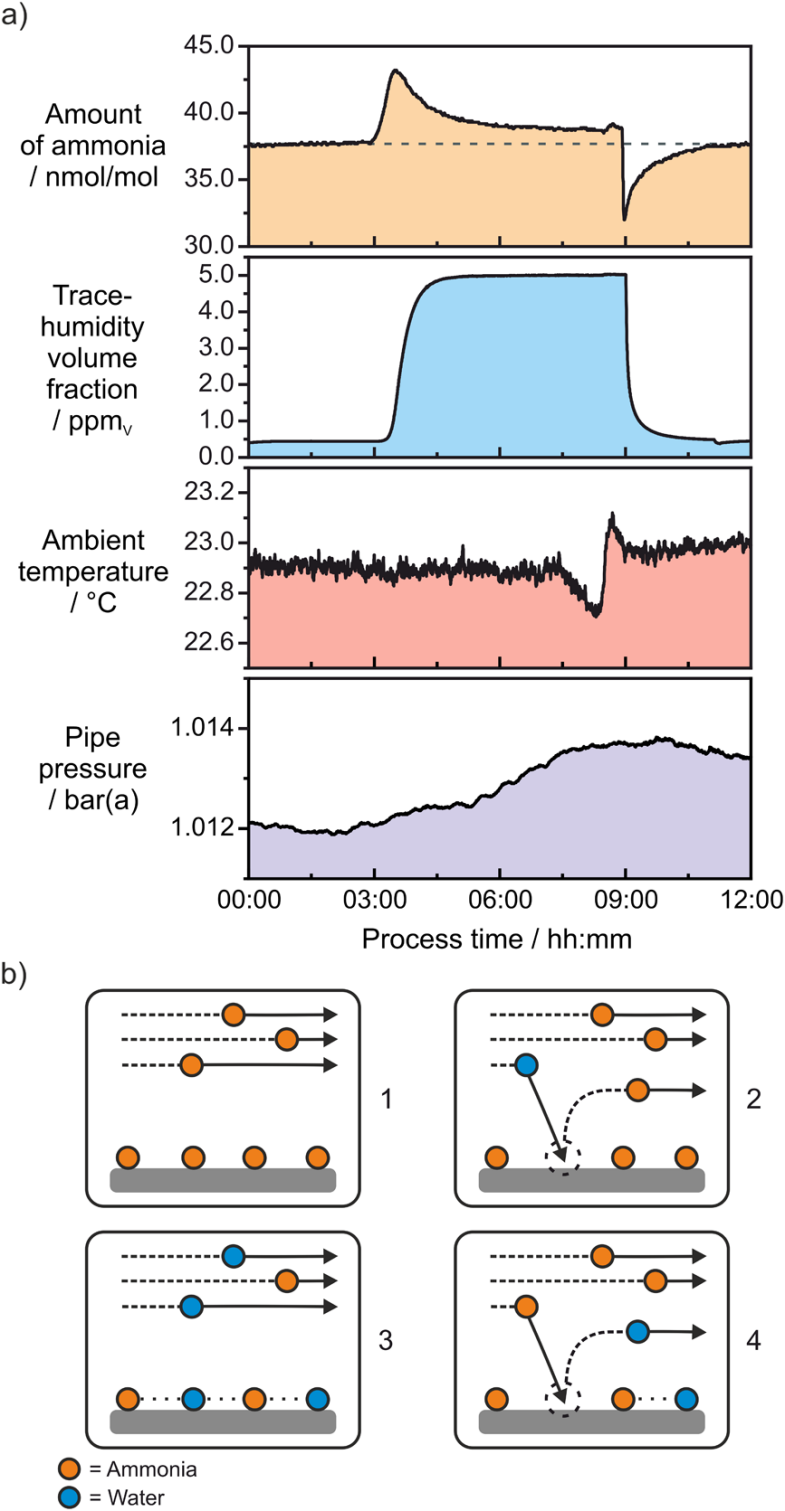
(a) Time series of ammonia, humidity, ambient temperature, and pipe pressure readings. The data set covers a 3-h dry prephase, followed by a 6-h humidification phase (5.01 ± 0.03) ppm_V_, and concludes with a 3-h dry postphase. (b) Illustration of surface dynamics: (1) equilibrium between adsorbed and free ammonia, (2) ammonia desorption triggered by water adsorption, (3) establishment of a steady state where water is adsorbed in the presence of ammonia, and (4) thermodynamically driven water desorption, leading to surface sites that result in ammonia adsorption.

The pipe pressure remained near atmospheric levels for all measurements at an average of (1.016 ± 0.016) bar­(a), while the laboratory temperature was maintained at an average of (22.8 ± 1.4) °C by the air conditioning system. The presented time series shows minor ambient temperature fluctuations of ± 0.2 K between 7:30 and 9:00 h of process time. This oscillation occurred irregularly and rarely, was attributed to the air conditioning system, and is presented here for the sake of completeness.

In the dry prephase of the time series, the amount of ammonia was found to slightly vary at around 37.8 nmol/mol, while the trace-humidity volume fraction remained at 0.44 ppm_V_. This clearly indicates that the inner surface of the piping was in equilibrium between adsorbed and free ammonia, as illustrated in Figure 2b-1. However, it is unlikely that all adsorbed ammonia is directly adsorbed on the bare steel surface, as a monolayer of water is expected to be present, a phenomenon that is supported by the literature.^58–62^ In our study, during dry pre- and postphase, the partial pressure of water vapor was less than 0.0018 % of its saturation level, which, according to the literature, indicates the presence of a so-called ″ice-like″ water monolayer on the piping surface, characterized by structural ordering and constrained molecular dynamics.^63,64^

During the humidification phase of (5.01 ± 0.03) ppm_V_, the amount of ammonia initially rose from 37.8 nmol/mol to a maximum peak of 43.2 nmol/mol before gradually decreasing to a new baseline of 38.8 nmol/mol, which is 1.0 nmol/mol higher than the initial amount (quantitatively evaluated below). The first observation of the desorption of adsorbed ammonia aligns with literature findings, which clearly show that water is a stronger adsorbent than ammonia, as illustrated in Figure 2b-2.^65–67^

When switching back, the system shifts from a thermodynamically stable state of adsorbed ammonia in the presence of water (Figure 2b-3) to a fully dry phase. There, the amount of ammonia decreased rapidly and sharply to a minimum of 32.0 nmol/mol, followed by a gradual increase back to the baseline amounts of the prephase. This can be explained by the desorption of adsorbed water, as the equilibrium between adsorbed and free water shifts abruptly, thereby favoring desorption, according to Le Chatelier’s principle (Figure 2b-4). In this process, surface sites are vacated, which are rapidly occupied by ammonia. Consequently, the amount of gas-phase ammonia decreased. With increasing saturation of the surface sites with ammonia, the system gradually returned to its equilibrium dry state with 37.8 nmol/mol (Figure 2b-1). These observations are consistent with a physisorption-dominated process, although chemisorption sites cannot be ruled out.

### Measurement Bias

The observation of a new elevated baseline in the trace-humidified state was unexpected. Nevertheless, this phenomenon occurred reproducibly during all 79 measurements in trace-humidified hydrogen, independently of the investigated amount of ammonia, as depicted in Figure 3a (see SUI Table S2). It is important to note that within this measurement campaign, individual measurement points were repeated at different timestamps (see SUI Table S1) to confirm reproducibility. The observed bias exhibited a nonlinear and positive increase. Subsequent data fitting resulted in a histogram of the residuals that closely followed a Gaussian distribution, as shown in Figure 3b. A more detailed discussion of this finding, along with the fitting procedure, is presented below.

**Figure 3:**
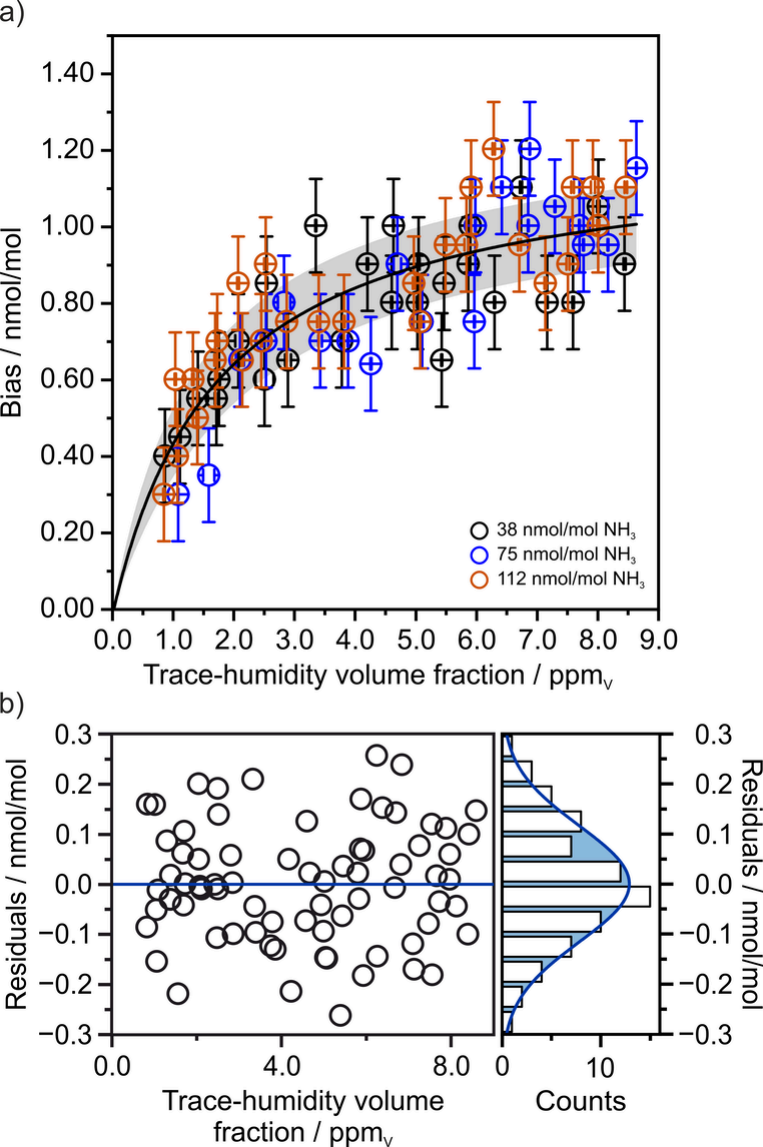
Observed ammonia biases in the trace-humidified states were plotted as a function of the humidity volume fractions and subsequently analyzed. In (a), all recorded biases are plotted and modeled using a Langmuir-like isotherm. The confidence interval (gray shaded area) reflects the uncertainty of the model fit, indicating the range in which the true mean response is expected to lie with 68% confidence. (b) shows the residuals of the fit along with a histogram, indicating that the residuals are normally distributed (bin width: 0.05 nmol/mol).

Initially, cross-sensitivity of the ammonia absorption band to water was suspected. Ammonia absorption bands around 1500 nm^68–70^ and 2200 nm^71–74^ have been reported as analyte specific. Note here that the exact ammonia absorption band studied here remains proprietary information of AP2E but falls within the range of (2.2 ± 0.2) μm, placing it within the near-infrared (NIR) spectrum. However, as demonstrated in the SUI, no spectral interferences were observed within the ammonia absorption band when comparing the spectra of trace-humidified hydrogen without ammonia and trace-humidified hydrogen with ammonia to that of ammonia in dry hydrogen. To further validate this, we directly measured the laser emission used for ammonia quantification with an optical spectrum analyzer, thereby accurately determining the spectral window of the laser. We compared the water absorption spectrum from the HITRAN^75^ database within the quantified spectral window of the ammonia laser (see SUI Figure S2). As a result, no significant water absorption bands were identified that could prove cross-sensitivity within the investigated measurement range. Therefore, no evidence of cross-sensitivity was found within the resolution of the system.

Another possible explanation for the elevated baseline is the continuous desorption of adsorbed ammonia into the gas phase. This would require that the total negative integral of desorbed ammonia in the humidified state equals the corresponding integral in the dry state, in accordance with mass conservation principles and under the assumption of a constant number of surface adsorption sites, as already presented in the literature.^65,66^ However, integration clearly shows that this is not the case here – a conclusion that is also readily apparent from a cursory visual examination of our data. To maintain consistency, the integral in the humidified phase was calculated using the elevated baseline. With this approach, the integrals of ammonia desorption and adsorption are in agreement.

Furthermore, our OF-CEAS system is able to quantify multiple gaseous components, such as carbon monoxide, carbon dioxide, and methane, in addition to ammonia. However, the observed trace-humidity-induced effect was exclusively observed for ammonia. This indicates the presence of a systematic bias that is specific to ammonia. To better understand the observed biases, the results were evaluated within the framework of the Beer–Lambert law:

$$E_{\lambda }=\log_{10}\left(\frac{I_{0}}{I}\right)=c\cdot \varepsilon \cdot d$$ 1

where *E*_λ_ is the absorbance, *I*_0_ the intensity of the incident light, *I* the intensity of the transmitted light, *c* the concentration, ε the decadic extinction coefficient, and *d* the OPL. Therefore, it becomes evident that the absorbance of ammonia has increased. Given that in the trace-humidified state the amount of ammonia was theoretically reduced by a maximum of only 0.08%, corresponding to a decrease of only 0.03 nmol/mol from an initial amount of 37.8 nmol/mol, this change falls below the resolution of the OF-CEAS. Consequently, from a metrological perspective, the supplied amount of ammonia can be considered constant under both dry and humidified conditions. Moreover, the OPL remained unchanged because it is a fixed device parameter (validated – photon lifetime remained constant).

Therefore, a possible explanation for the observed increase in absorbance could be a variation in the decadic extinction coefficient, an intrinsic property that quantifies the strength of light absorption by a substance at a specific wavelength. It is known to be influenced by various factors, such as pH and ionization state,^76–78^ as well as solvent effects.^79–81^ In the gas phase, such variations would necessitate the formation of gaseous ammonia–water clusters. However, under the experimental conditions (gas cell pressure of 100 mbar­(a) and temperature of 45 °C), the formation of such clusters is unlikely. Furthermore, if gas phase clustering were a dominant effect, substantially larger measurement deviations would be expected, as the amount of trace humidity significantly exceeded that of ammonia.

However, this assumption does not apply to ammonia adsorbed on the hyperreflective mirrors of the gas cell. According to the literature, the presence of ammonium in water monolayers has been experimentally confirmed using surface spectroscopic methods.^82,83^ Furthermore, Lechner et al. observed ammonium adsorbed on Pt(111) using Scanning Tunneling Microscopy (STM).^60^ Therefore, the introduction of trace humidity will likely facilitate the formation of ammonium on the surface of the mirrors, shifting the surface pH toward alkaline conditions. Additionally, ammonia solvation in water monolayers or the formation of ammonia–water clusters on the surface may influence the absorbance through solvation effects, such as hydrogen bonds.^84,85^ Literature also reports how ammonium (ammonia hydrates) exhibits significant spectral absorbance variations compared to ammonia.^86–88^ The interaction of potential ammonium species on or with the surface, probed by the oscillating laser over an OPL of 6.17 km, may interfere with the absorption signal. However, it should be noted that the literature reports line broadening of ammonia absorption bands at 1512.2 and 1516.0 nm under humidified conditions.^89^ Therefore, determining the integrated absorbance using a constrained fitting approach with reduced degrees of freedom, as performed here, would lead to a negative bias. While this could mimic a change in the extinction coefficient, the effect arises solely from model underfitting rather than from actual spectroscopic changes. Consequently, we assume that this is not the observed effect that results in a positive bias.

As stated above, the supplied amount of ammonia remained constant in both the dry and humidified phases. However, it is important to distinguish between the supplied and accumulated ammonia in the trace-humidified phase. If Figure 3a is interpreted as an adsorption isotherm, it effectively describes how the amount of ammonia adsorbed on the surface of the hyperreflective mirrors increased with rising trace-humidity volume fractions. Consequently, the data were fitted using a Langmuir-like isotherm model:

$$\Gamma_{e}=\frac{\Gamma_{m}\cdot K_{\mathrm{eq}}\Phi_{w}}{1+K_{\mathrm{eq}}\Phi_{w}}$$ 2

where Φ_w_ represents the trace-humidity volume fractions, Γ_e_ the equilibrium surface coverage, Γ_m_ the maximum surface coverage, and *K*_eq_ the equilibrium constant.^58^ The estimated parameters were Γ_m_ = (1.21 ± 0.05) nmol/mol and *K*_eq_ = (0.57 ± 0.08) ppm_V_^–1^. For completeness, it should be noted that three mirrors were mounted in the measuring cell (V-shaped cavity), each with a surface area of approximately 0.39 cm^2^ (diameter of approximately 0.7 cm, see SUI). However, Figure 3b shows that the histogram of the Langmuir-like isotherm demonstrates a normally distributed variance, indicating an accurate average description of the data. Furthermore, the Shapiro-Wilk test yielded a *p*-value of 0.906, which indicates a lack of evidence against the assumption that the data are normally distributed.^90^ Therefore, the introduction of trace humidity likely enriched the mirror surfaces with adsorbed water, facilitating the accumulation of free ammonia within a water-enriched layer (probed by the oscillating laser), which led to increased ammonia readings, observed as an increased baseline. Consequently, these elevated readings serve as an indirect measure of water adsorption. Moreover, the correlation between ammonia capture and surface water content has been well-documented in the literature, even at the level of water monolayers.^91–94^

Regardless of whether the described effects are the primary cause or whether an unexamined factor contributes to the observed bias, it is highly plausible that the observed effect originates from mirror interactions. To the best of our knowledge, such an effect has not been previously reported in the context of (ultra)­long-path OF-CEAS-based trace ammonia analysis. Furthermore, since our results demonstrate a fundamental effect, it can be assumed that this phenomenon is not specific to OF-CEAS as a measurement technique. Rather, similar effects are expected to occur when using other optical gas analysis methods, such as Cavity Ring-Down Spectroscopy (CRDS) or Off-Axis Integrated Cavity Output Spectroscopy (OA-ICOS). Although a deeper mechanistic investigation was not the target of this study, our data clearly demonstrate and quantitatively characterize a systematic, nonlinear, and humidity-dependent positive measurement bias across ammonia amounts relevant to hydrogen purity analysis. These findings substantiate the need to adapt the uncertainty budget in trace ammonia analysis when using an (ultra)­long-path optical gas measurement system. In addition to well-known instrumental uncertainty sources, such as standard deviation, repeatability, calibration accuracy, and instrument drift, the influence of trace humidity must be explicitly included as a systematic effect. This further highlights the importance of simultaneous trace-humidity quantification as a direct influencing factor for reliable ammonia quantification.

### Adsorption & Desorption Kinetics

Qualitative surface dynamics and precisely defined measurement biases have been discussed above. In this section, a more detailed investigation into the transition states from a dry phase to a trace-humidified phase, as well as the reverse transition from a trace-humidified to a dry phase, will be explored. The transition from a humidified to a dry state is discussed first, as the obtained experimental results provide a better understanding of the reverse transition.

In this context, we would like to highlight two important points. First, the data presented here were collected over a period of four months, during which the measurements showed consistent reproducibility. All measurement results and relevant parameters, including timestamps, are provided in the SUI Table S1. Second, we would like to clearly point out that the reaction rates discussed below correspond to initial reaction rates, as the system undergoes a transition from a thermodynamically stable state to a dynamic regime.

Generally, the adsorption of ammonia from the gas phase proceeds in two sequential steps. First, water desorption from the surface (according to Le Chatelier’s principle) occurs, creating an available adsorption site:

$$H_{2}O_{\left(\mathrm{ad}\right)}\to \mathrm{site}+H_{2}O_{\left(g\right)}↑$$ 3

Here, (ad) denotes the adsorbed phase and (g) represents the gas phase. In our interpretation, the specific nature of the adsorption site – whether located on a water film or directly on the metal surface – is not distinguished. Subsequently, free ammonia molecules adsorb onto the exposed site:

$${\mathrm{NH}}_{3(g)}+\mathrm{site}\to {\mathrm{NH}}_{3(\mathrm{ad})}$$ 4

Because these two processes occur concurrently, we can simplify the investigated initial reaction as follows:

$${\mathrm{NH}}_{3(g)}+H_{2}O_{\left(\mathrm{ad}\right)}\to {\mathrm{NH}}_{3(\mathrm{ad})}+H_{2}O_{\left(g\right)}$$ 5

Consequently, the rate law for the overall reaction can be described as follows:

$$r=k\cdot [{\mathrm{NH}}_{3(g)}{]}^{n}[H_{2}O_{\left(\mathrm{ad}\right)}{]}^{m}$$ 6

where *r* is the reaction rate, *k* the reaction rate constant, *n* is the reaction order with respect to ammonia and *m* the reaction order with respect to adsorbed water. Given that (initial) adsorption rates of ammonia were investigated as a function of trace-humidity volume fractions, while the amount of supplied ammonia remained constant, we simplify the rate law (eq 6) to

$$r=k_{1}^{\prime }\cdot [H_{2}O_{\left(\mathrm{ad}\right)}{]}^{m}$$ 7

with

$$k_{1}^{\prime }=k\cdot [{\mathrm{NH}}_{3(g)}{]}^{n}$$ 8

where *k*_1_^’^ is the pseudo rate constant. Since the investigated trace-humidity volume fractions remained below a partial pressure of 0.90 Pa, and reversible processes were observed, we apply the Langmuir adsorption isotherm, which describes an approximately linear relationship between the amount of gas adsorbed and its partial pressure at low pressures.^58,95^ Consequently, we simplify with

$$\left[H_{2}O_{\left(\mathrm{ad}\right)}]\propto [H_{2}O_{\left(g\right)}\right]$$ 9

to the final form of the rate law:

$$r\propto k_{1}^{\prime }\cdot [H_{2}O_{\left(g\right)}{]}^{m}$$ 10

Applying the natural logarithm to this rate law yields

$$\ln(r)\propto \ln(k_{1}^{\prime })+m\cdot \ln([H_{2}O_{\left(g\right)}])$$ 11

as plotted in Figure 4a. In this logarithmic plot, the slope of the linear regression corresponds to the reaction order *m* (with respect to water) and the intercept provides an estimate of the pseudo rate constant. The parameters are listed in Table 1.

**Figure 4:**
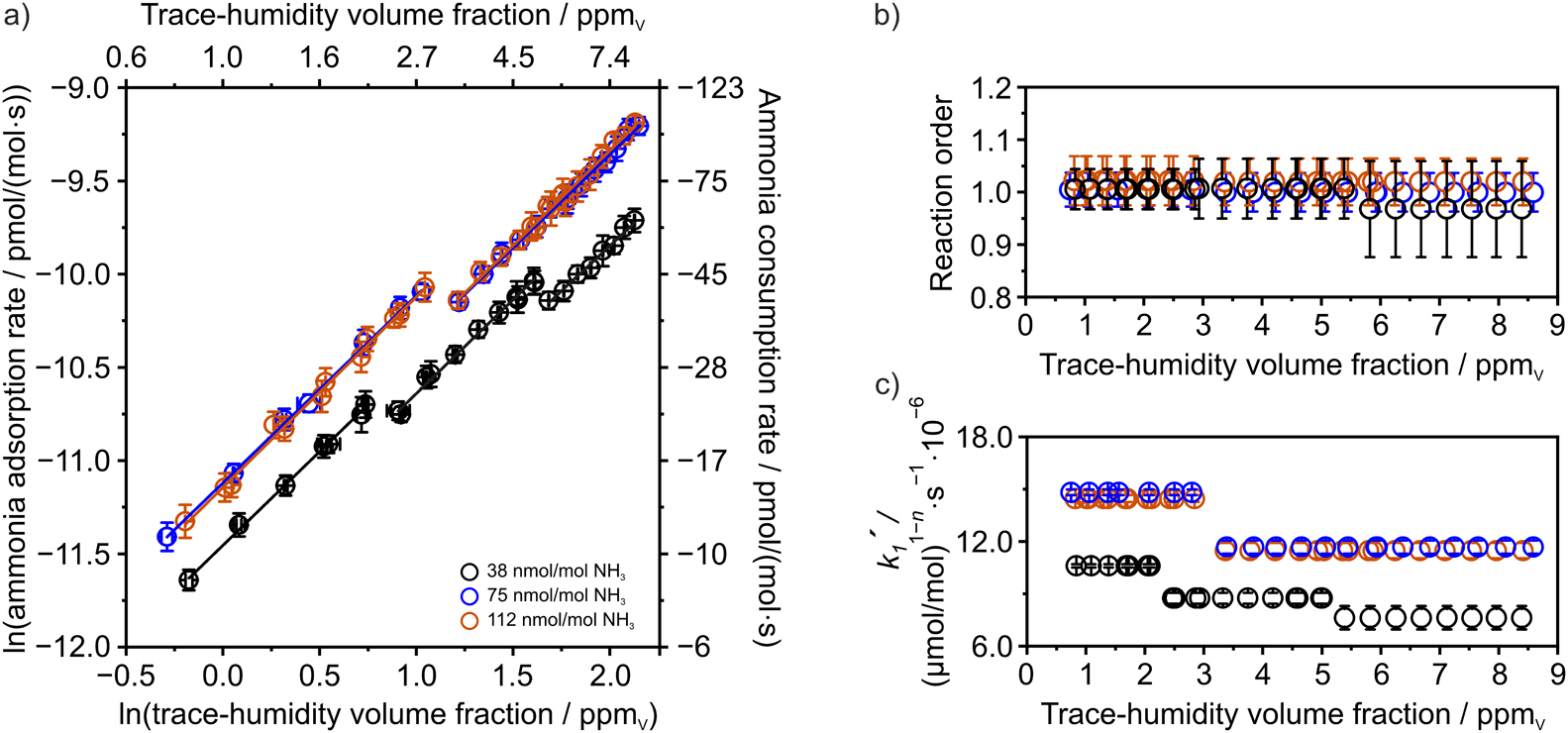
Illustration of experimentally derived kinetic data for the transition from a trace-humidified state to a dry state. (a) Displays the measured rates of free ammonia consumption and ammonia adsorption as a function of the investigated trace-humidity volume fractions. The presence of multiple linear ranges for the three examined amounts of trace ammonia indicates distinct solvation states for the adsorbed ammonia. The intercept of the natural logarithmic linear regression represents the pseudorate constant, whereas the slope indicates the reaction order for the humidity desorption. (b) Depicts the estimated reaction order for humidity desorption, and (c) illustrates the estimated pseudorate constants for humidity desorption. Please note that the legend color coding in (a) also applies to panels (b) and (c).

**Table 1:** Listing of the Determined Reaction Orders and Pseudo Rate Constants for Water Desorption during the Transition from a Humidified to a Dry State^a^

amount of ammonia (nmol/mol)	trace-humidity range (ppm_V_)	reaction order (*m*)	*k*_1_^′^/(μmol/mol)^1 – *n*^ · s^–1^ · 10^–6^
38.2	<2.5	1.01 ± 0.04	10.60 ± 0.01
	>2.5 to approximately 5.0	1.01 ± 0.06	8.75 ± 0.31
	>5.0	0.97 ± 0.09	7.63 ± 0.67
74.8	<3.0	1.00 ± 0.03	14.82 ± 0.02
	>3.0	1.00 ± 0.04	11.67 ± 0.04
112.1	<3.0	1.02 ± 0.05	14.46 ± 0.02
	>3.0	1.02 ± 0.04	11.48 ± 0.04

a The uncertainty of the parameters is specified with a coverage factor of 95%.

Figure 4a shows the (initial) consumption rates of gas-phase ammonia in the transition phase from humid to dry as a function of the investigated trace-humidity volume fractions. These were obtained by plotting a linear regression of the data points starting from the onset of the abrupt decrease in ammonia readings, which exhibited a strongly linear trend until the minimum value was observed just before the recovery phase started, during which the readings returned to the baseline amounts established in the prephase (Figure 2a). An example of a linear fit, along with a comprehensive list of all determined rates, is provided in the SUI. The obtained consumption rates revealed the presence of distinct linear regression ranges. For ammonia amounts of 112 nmol/mol and 75 nmol/mol, both exhibit a strong agreement, showing an initial linear range up to 3 ppm_V_, followed by a second linear range beyond 3 ppm_V_. Interestingly, the rates for 38 nmol/mol show three distinct linear ranges. The first range extends up to 2.5 ppm_V_, the second spans from 2.5 ppm_V_ to approximately 5 ppm_V_, and the third begins beyond 5 ppm_V_. Furthermore, by changing the sign of the (initial) consumption rates, (initial) ammonia adsorption rates were obtained, which were used to apply eq 11.

Figure 4b shows the reaction order of water desorption. As the system changes from a humidified to a dry state, desorption is driven by the disruption of the thermodynamic equilibrium, leading to the release of adsorbed water into the gas phase. Our results clearly demonstrate that water desorption consistently follows first-order kinetics. Furthermore, our experimental results align with theoretical findings which describe that the desorption of an individual water molecule follows a first-order reaction, whereas the subsequent rearrangement of the sorption layer, involving the formation of new hydrogen bonds, follows higher-order kinetics.^96,97^

Figure 4c shows the pseudo rate constants, in which a slowdown of the water desorption process is observable. On a macroscopic level, the slowdown implies that water exhibits stronger adhesion to the surface. Furthermore, considering that the adhesive property of adsorbed water further increases with increasing trace-humidity volume fractions, the formation of thermodynamically more stable ammonia–water clusters could be considered. Moreover, as already discussed and supported by literature findings, we assume the presence of ammonium on the surface, making the discussion of protonated ammonia–water clusters particularly relevant. Several studies have investigated the formation and stability of protonated ammonia–water clusters, examining their dependence on cluster size and structural configuration. These studies consistently demonstrate that ammonia preferentially forms stable clusters with water.^98–101^ Moreover, Oostenrijk et al. reported that the stability of ammonia–water clusters increases with the addition of water molecules.^102^ Their theoretical findings show that the dissociation energy between ammonia and the surrounding water environment is enhanced, leading to greater cluster stability as the energy required to break the hydrogen-bonded network increases. Furthermore, they reported that water molecules contribute to charge stabilization by mitigating Coulombic repulsion within the cluster. The authors validated their theoretical insights experimentally through mass spectrometry, which revealed that increased solvation enhances structural integrity, reduces fragmentation, and improves overall cluster stability.^102^

Considering that the actual water desorption follows first-order kinetics, the rate-determining step for the desorption process appears to be the breakdown of a surface-associated hydrogen-bonded ammonia–water network to release a water molecule. This breakdown is likely limited due to rotational energy barriers, which must be overcome before water molecules can desorb from the sorption layer. After water desorption, a rearrangement of the remaining ammonia–water network is assumed, likely following higher-order kinetics. In conclusion, our experimental findings reveal distinct hydration states of ammonia on surfaces, which validate and substantiate various findings from the literature. Furthermore, our data suggest that a higher degree of hydration states is present at 38 nmol/mol than at the other two amounts studied, based on the presence of three distinct ranges and significantly lower pseudo rate constants observed. This is most likely due to reduced competition for adsorbed water at lower amounts of ammonia, allowing for more extensive hydration states.

In the following part, the transition phase from dry to trace-humidified conditions is discussed. The water-induced desorption process of adsorbed ammonia into the gas phase is likely more complex. However, its initial reaction can be described by the following equation:

$${\mathrm{NH}}_{3(\mathrm{ad})}+H_{2}O_{\left(g\right)}\to {\mathrm{NH}}_{3(g)}+H_{2}O_{\left(\mathrm{ad}\right)}$$ 12

The rate law for the overall reaction can be described as follows:

$$r=k\cdot [{\mathrm{NH}}_{3(\mathrm{ad})}{]}^{y}[H_{2}O_{\left(g\right)}{]}^{z}$$ 13

where *y* is the reaction order with respect to adsorbed ammonia and *z* the reaction order with respect to water vapor. As already applied above, the pseudo rate constant is used here with

$$k_{2}^{\prime }=k\cdot [{\mathrm{NH}}_{3(\mathrm{ad})}{]}^{y}$$ 14

The corresponding pseudo rate equation can be simplified as

$$r=k_{2}^{\prime }\cdot [H_{2}O_{\left(g\right)}{]}^{z}$$ 15

and the natural logarithm of this pseudo rate equation takes the form

$$\ln(r)=\ln(k_{2}^{\prime })+z\cdot \ln([H_{2}O_{\left(g\right)}])$$ 16

as plotted in Figure 5a. The parameters are listed in Table 2.

**Figure 5:**
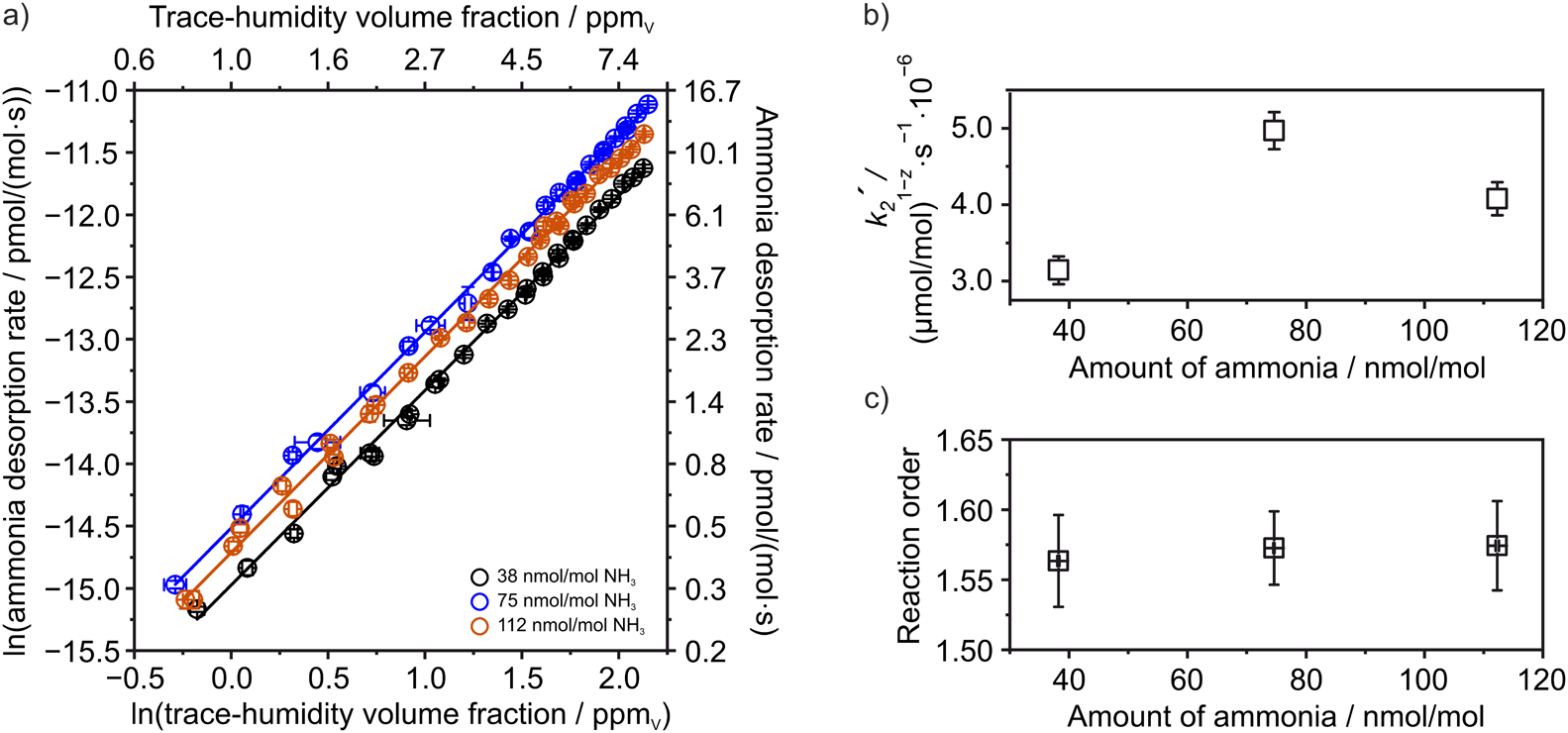
Visualization of experimentally derived kinetic data describing the transition from a dry state to a trace-humidified state. (a) Shows the measured desorption rates of absorbed ammonia as a function of the investigated trace-humidity volume fractions. The intercept of the natural logarithmic linear regression represents the pseudorate constant, whereas the slope indicates the reaction order for humidity adsorption. (b) Estimated pseudorate constants for humidity adsorption, and (c) illustration of the corresponding reaction order.

**Table 2:** An Overview of the Experimentally Determined Reaction Orders and Pseudo Rate Constants for the Trace-Humidity-Induced Desorption of Ammonia^a^

amount of ammonia (nmol/mol)	reaction order (*z*)	*k*_2_^′^/(μmol/mol)^1 – *z*^ · s^–1^ · 10^–6^
38.2	1.56 ± 0.03	3.14 ± 0.15
74.8	1.57 ± 0.03	4.97 ± 0.20
112.1	1.57 ± 0.03	4.08 ± 0.18

a With uncertainties reported at a coverage factor of 95%.

Figure 5a illustrates the ammonia desorption rates corresponding to the increase in gas phase ammonia readings upon introducing trace humidity into the system. The desorption rates were estimated by applying a linear regression to the rising phase of the readings (SUI). The initial fitting point was chosen approximately at the elevated baseline level. Within each measurement cycle, the ammonia desorption rates showed a nonlinear upward trend, becoming steeper at higher trace-humidity volume fractions (for better illustration, see SUI Figure S5).

The observed water adsorption pseudo rate constants are plotted in Figure 5b and illustrate that the humidity adsorption accelerates by approximately 58% as the amount of ammonia increases from 38.2 nmol/mol to 74.8 nmol/mol. However, for the further increase from 74.8 to 112.1 nmol/mol, the humidity adsorption slows down by 18%. Therefore, from a macroscopic perspective, it indicates that water initially displaces ammonia from the surface more effectively, but at 112.1 nmol/mol, a barrier emerges. Also, it should be noted that significantly larger quantities of water/trace humidity were present than ammonia throughout the humidified measurement cycles. Therefore, considering the aforementioned reversible characteristics, we can only conclude that at higher trace amounts of ammonia, stronger electrostatic interactions lead to stronger ammonia adhesion to the sorption layer/quasi-static surface layer. This interpretation is supported by numerous theoretical and experimental literature studies demonstrating that ammonia forms hydrogen-bonded clusters through intermolecular interactions.^103–106^ These studies have often highlighted two key aspects: first, the structures of cyclic ammonia clusters; and second, the distinction between neutral and protonated ammonia clusters.^85,107–109^ While our approach does not allow for precise determination of transitions between specific cluster states, we were able to identify and describe this macroscopic phenomenon by interpretation of our results.

Figure 5c shows the reaction orders that were observed to be 1.57 ± 0.03 for the adsorption process with respect to water. A fractional reaction order indicates a more complex reaction mechanism and is often described for surface reactions.^110–113^ As previously discussed, we must assume that ammonia was bound to a water layer. Lechner et al. reported that the adsorption of ammonia occurs on a second layer of water molecules, where the water molecules located within this layer reorient to expose a dangling H, pointing to the ammonia and forming a hydrogen bond.^60^ Consequently, to enable desorption, these specific hydrogen bonds must be overcome in order to release ammonia from the surface while new bonds will form with adsorbing water. If ammonia undergoes additional electrostatic coordination through intermolecular interactions with other ammonia molecules (as interpreted above), the adsorbing water molecule must overcome a higher energy barrier to induce ammonia desorption. While this increased barrier likely explains the observed deceleration in reaction kinetics, the overall (apparent) reaction mechanism seems to remain unchanged, as it can be derived from the results of our measurements and method. Based on these observations, we conclude that the water adsorption mechanism follows a mixed-order kinetic model, where the rate-determining step involves the breaking and rearrangement of surface van-der-Waals interactions to facilitate the release of ammonia into the gas phase.

In summary, whereas Figure 3a describes thermodynamically stable humidification states as biases using a Langmuir-like isotherm, this section describes the transition states from a macroscopic perspective. This allows us to characterize water–ammonia interactions not only at thermodynamic equilibrium but also within a dynamic regime through the analysis of reaction kinetics. Furthermore, the experimental data presented here can provide a valuable foundation for research groups working on molecular modeling of ammonia–water interface systems.

### Reactivity

As outlined in the previous sections, we assume that ammonia exists as ammonium in its adsorbed state to a certain extent. If this hypothesis proves valid, it implies the presence of ammonium hydroxide, a base with distinct reactivity properties. One of the simplest reactions that ammonium hydroxide can undergo is with carbon dioxide to form ammonium bicarbonate:

$${\mathrm{NH}}_{4}\mathrm{OH}+{\mathrm{CO}}_{2}⇋({\mathrm{NH}}_{4}){\mathrm{HCO}}_{3}$$ 17

Ammonium bicarbonate is a thermally unstable compound with a decomposition vapor pressure. Applying the Clausius–Clapeyron equation with an estimated sublimation enthalpy of 68.8 kJ/mol,^114^ the vapor pressure of ammonium bicarbonate at 23 °C is calculated to be 79.9 mbar­(a). Therefore, the conversion of ammonium hydroxide to ammonium bicarbonate generates a dissociation equilibrium, releasing ammonia, water, and carbon dioxide:

$$({\mathrm{NH}}_{4}){\mathrm{HCO}}_{3}⇋{\mathrm{CO}}_{2}+H_{2}O+{\mathrm{NH}}_{3}$$ 18

Consequently, in an ammonia-containing gas mixture, this dissociation process would contribute an additional partial pressure of ammonia, which simultaneously increases the total amount of ammonia in the system.

Using this approach, we conducted the measurement, with a corresponding time series presented in Figure 6. The time series consisted of a 2-h prephase, main phase, and postphase, with carbon dioxide being introduced in the main phase. During the prephase, the amount of ammonia was 111.3 nmol/mol, the trace-humidity volume fraction was 346.2 ppb_V_, and the carbon dioxide blank value was 80.0 nmol/mol.

**Figure 6:**
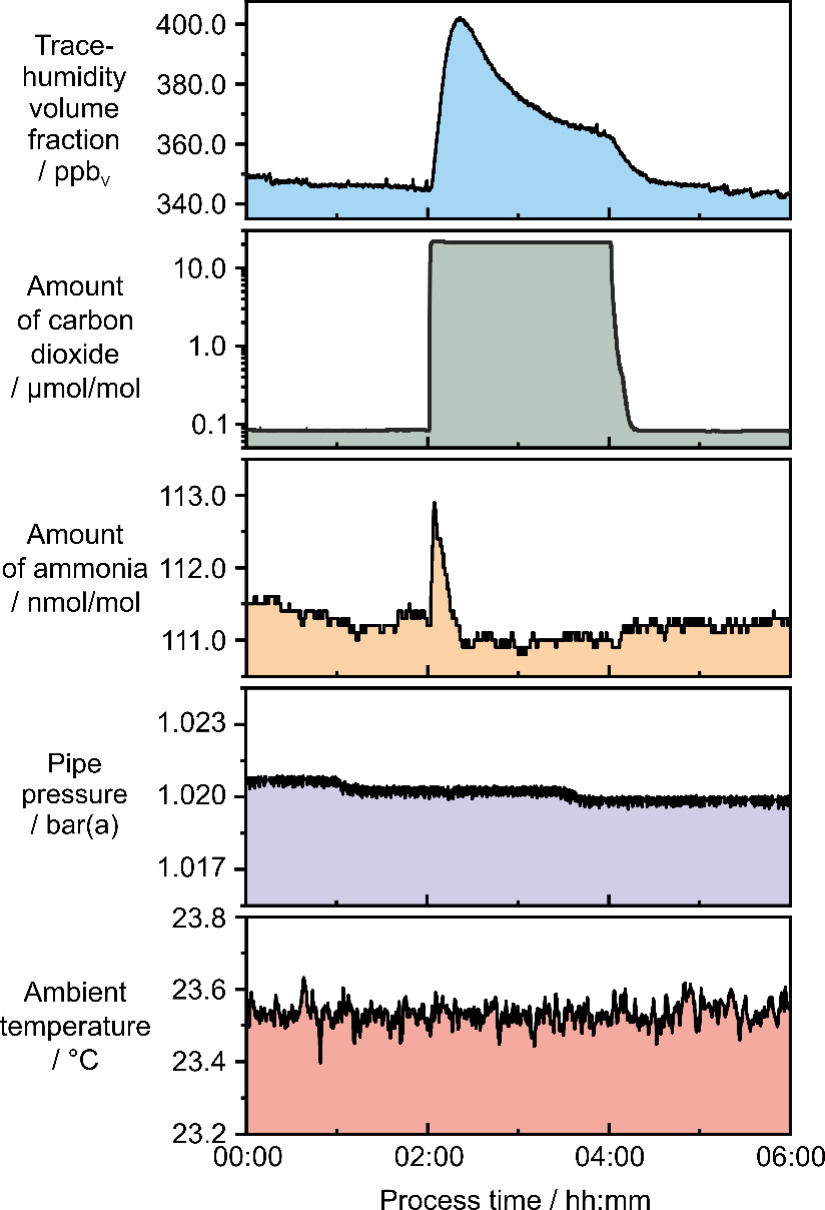
Time-resolved response of ammonia-containing dry sample gas to a controlled carbon dioxide pulse. Throughout the experiment, the laboratory temperature remained stable at (23.55 ± 0.05)°C and the line pressure at (1.020 ± 0.001) bar­(a). Significant water desorption was observed when carbon dioxide was injected. In summary, the data show that a carbon dioxide pulse triggers an irreversible release of adsorbed water and a short but irreversible release of adsorbed ammonia from the piping system.

Transitioning from the prephase to the main phase, the amount of carbon dioxide increased to a reading of 21.0 μmol/mol. Theoretically, dynamic dilution of the carbon dioxide-containing CRM into the sample gas reduces the amount of ammonia by 0.22%, corresponding to an absolute decrease of 0.24 nmol/mol. This further dilution was found and reflected in the ammonia readings, which dropped to 111.0 nmol/mol. However, this also implies that a slightly negative bias was induced during the transition from the prephase to the main phase. Despite this, it is clearly observable that upon the introduction of carbon dioxide, the ammonia reading spikes to a value of 112.9 nmol/mol, indicating a maximum increase of 1.6 nmol/mol. It is important to note that this desorption process is neither thermally nor pressure induced: the ambient temperature in the laboratory fluctuated around (23.55 ± 0.05) °C, and the line pressure remained almost constant around 1.02 bar­(a). After 20 min, the ammonia readings returned to the prephase baseline level, corresponding to a total desorbed mass of approximately 0.15 μg (8.8 nmol). Simultaneously, the trace-humidity readings spiked to 400 ppb_V_ and gradually declined over the course of the measurement. This corresponds to a total desorbed water mass of approximately 47 μg (2.6 μmol) and clearly shows that significantly larger quantities of water were desorbed from the piping system.

It could be hypothesized that the observed increase in humidity originates from the carbon dioxide-containing CRM. However, this hypothesis can be dismissed for several reasons: First, we quantified the trace-humidity volume fraction of the CRM to be less than 0.3 ppm_V_, which is negligible. Second, the lines and MFC connected to the CRM were thoroughly purged until trace-humidity volume fractions stabilized at approximately 350 ppb_V_. Moreover, if additional water had indeed been introduced into the system via the CRM­(-line), a decrease in ammonia readings would be expected during the transition from the main phase to the postphase, an effect that would be clearly visible with our equipment and has already been discussed in detail above. Therefore, as this scenario was not observed, we propose an entirely different surface mechanism. The water detected during the main phase likely originated from layers of adsorbed water within the piping system. Most likely, adsorbed water reacts with carbon dioxide to form carbonic acid, which in turn establishes a dissociation equilibrium, leading to continuous water desorption from the surface. These results provide further evidence that the assumption that ammonia adsorbs on a water film is well-founded and true, even if the trace-humidity level is kept at very low values.

The desorption of ammonia upon the introduction of carbon dioxide provides strong evidence that ammonium bicarbonate was most likely formed. However, a distinct and sharp desorption peak was only observed when a significant excess of carbon dioxide was introduced. At lower amounts, desorption effects were indeed still present but appeared less defined and rather as a broad peak. The sharp decline in ammonia desorption can be attributed either to the saturation of the product site in the dissociation equilibrium, thus inhibiting further dissociation, or due to depletion of the available or accessible ammonium ions within the sorption layer. Nevertheless, our data indirectly confirm the presence of ammonium on the sorption layer, supporting the validity of our previous assumptions.

Upon switching back to a carbon-dioxide-free gas mixture, all measured readings returned to their prephase amounts. However, as mentioned earlier, no decrease in the ammonia readings was observed, in contrast to the transitions involving trace humidity. This indicates that the process is irreversible, pointing toward a chemisorptive behavior, and that ammonia was not bound to a simple adsorption site. Furthermore, the thinning of the water film likely led to the degradation of the active chemisorptive sites. This observation further supports the presence of ammonium species on the surface.

## Conclusions

This study demonstrates that the quantification of trace amounts of ammonia using (ultra)­long-path OF-CEAS can be biased by surface adsorption processes in the presence of even trace amounts of humidity. Although the experiments were conducted using hydrogen as a matrix gas for purity analysis, our results justify the assumption that the observed phenomena and resulting insights are generally applicable and not limited to hydrogen-based systems. By applying dynamic dilution of gravimetrically prepared reference gases and highly precise real-time ammonia quantification via OF-CEAS, we identified and quantified a nonlinear positive measurement bias. This bias is driven by humidity-induced ammonia accumulation on hyper-reflective mirrors and is further amplified by the number of optical reflections. These findings underpin the critical importance of accurately quantifying and controlling trace-humidity levels when measuring trace levels of ammonia.

We observed reversible yet complex adsorption–desorption dynamics between ammonia and water and identified the underlying reaction kinetics. Under trace-humidified conditions, distinct hydration states of the adsorbed ammonia were observed. Conversely, under dry conditions and with increasing amounts of ammonia, stronger intermolecular interactions between ammonia molecules increased their adhesion to the surface. Additionally, the reaction order of water adsorption and desorption was determined with high precision, providing experimental validation of theoretical predictions.

Furthermore, we investigated the chemical reactivity of ammonia and carbon dioxide to indirectly confirm the presence of surface-bound ammonium in the sorption layer. This finding not only highlights the intrinsic reactivity of ammonia with water but also demonstrates the additional chemical reactivity of ammonium hydroxide in the trace range.

Overall, this study provides a robust foundation for the optimization of analytical methods aimed to achieve highly precise and reproducible ammonia quantification. Specifically, for (ultra) long-path optical gas measurement instruments, our findings indicate that operating gas cells at elevated temperatures (>45 °C) is advisable to minimize water adsorption and the consequent accumulation of ammonia.

## Supplementary Material



## Notes

Humidification unit design and operation; ammonia absorption band characterization; OF-CEAS gas cell’s mirrors; adsorption and desorption rate determination; summary of measurement data; and bias analysis for humidified vs dry states (PDF)

## Nomenclature

*p*: Pressure
*E*_*λ*_: Absorbance
*I*_0_: Intensity of the incident light
*I*: Intensity of the transmitted light
*c*: Concentration
*ε*: Decadic extinction coefficient
*d*: Optical path length
Γ_e_: Equilibrium surface coverage
Γ_m_: Maximum surface coverage
*K*_eq_: Equilibrium constant
Φ_w_: Trace-humidity volume fraction
*r*: Reaction rate
*k*: Reaction rate constant
*n*: Reaction order with respect to gas-phase ammonia
*m*: Reaction order with respect to adsorbed water
*y*: Reaction order with respect to adsorbed ammonia
*z*: Reaction order with respect to water vapor
*k*_1_^’^, *k*_2_^’^: Pseudo rate constant
