# Supplementary material for: Trace-Level Ammonia–Water Interactions in Hydrogen: Challenges in Gas Purity Analysis Using Optical-Feedback Cavity-Enhanced Absorption Spectroscopy (OF-CEAS)
Source: ACS Meas Sci Au. 2025 Oct 13;5(6):897–911. doi: 10.1021/acsmeasuresciau.5c00105 (PMC12715633; doi:10.1021/acsmeasuresciau.5c00105)
Supplement: Supplementary file 1 [file tg5c00105_si_001.pdf]

# Supporting Information

## Trace-Level Ammonia–Water Interactions in Hydrogen: Challenges in Gas Purity Analysis Using Optical-Feedback Cavity-Enhanced Absorption Spectroscopy (OF-CEAS)

Mehmet Emin Bayat[1,2], Heinrich Kipphardt[1], Carlo Tiebe[1], Dirk Tuma[1] and Carsten Engelhard [1,2,*]

1) Bundesanstalt für Materialforschung und -prüfung (BAM), Richard-Willstätter-Str. 11, 12489 Berlin, Germany
2) Department of Chemistry and Biology, and Center of Micro- and Nanochemistry and (Bio-)Technology (Cμ), University of Siegen, Adolf-Reichwein-Str. 2, 57068 Siegen, Germany

*Email: Carsten.Engelhard@bam.de

ORCIDs: M. E. Bayat: 0009-0008-6883-4315, H. Kipphardt: 0009-0003-6081-8090, C. Tiebe: 0009-0009-8247-5440, D. Tuma: 0000-0002-5713-9746, C. Engelhard: 0000-0002-7020-9278.

Keywords: hydrogen, ammonia, humidity, OF-CEAS, adsorption, surface interactions.

# 1. Humidification unit

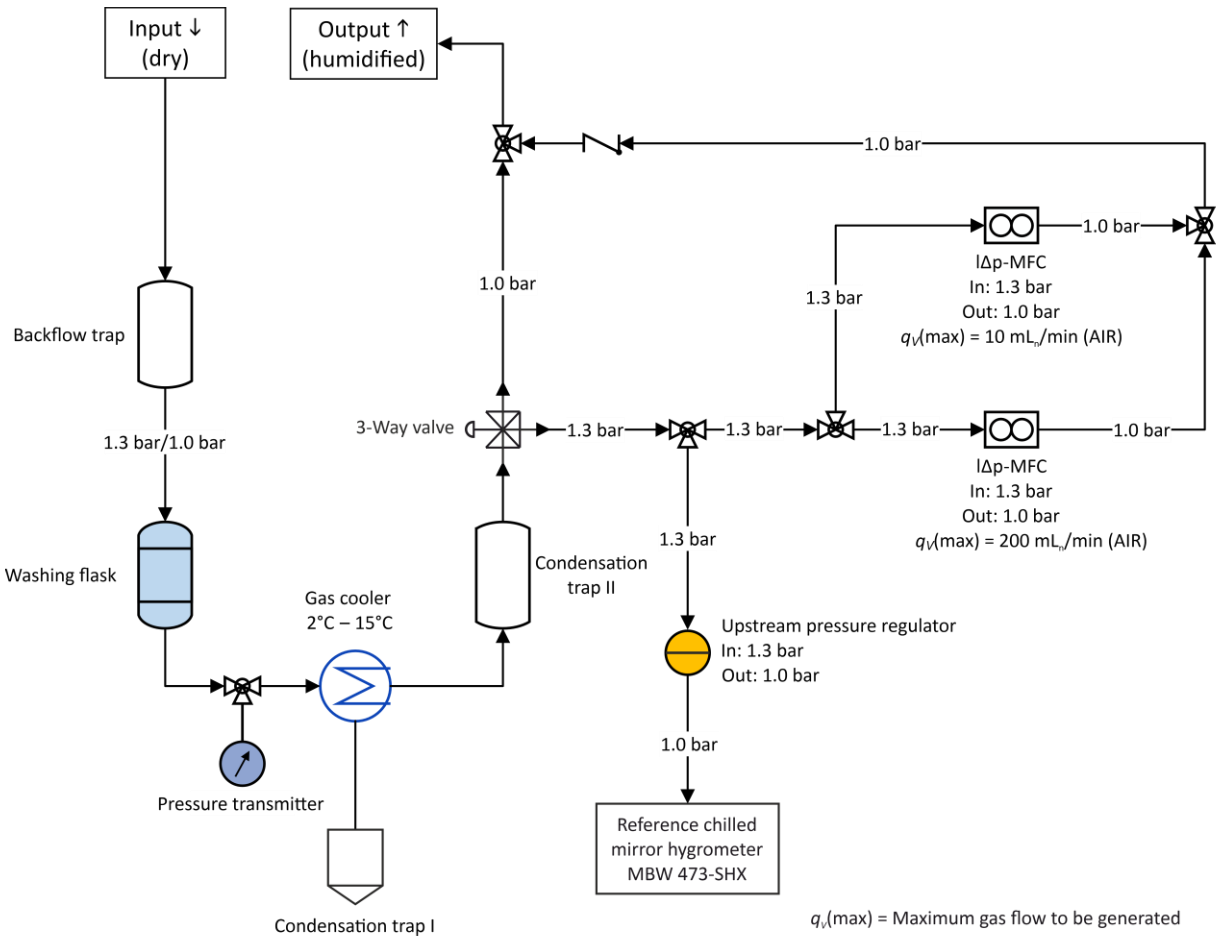


**Figure S1**: Schematic piping and instrument diagram of the humidification unit.

In Figure S1, a schematic piping and instrument diagram of the humidification unit (HU) is presented. A dry, particle-free hydrogen flow enters the washing flask (M&C TechGroup Germany, article number: 90F5305), where it undergoes saturation at a preset pressure (1.1 bar(a)–1.5 bar(a)) and ambient temperature. Upstream of this washing flask, a backflow trap (M&C TechGroup Germany, article number: 90F5305) is preventing water ingress into the piping system in the event of backflow. Following room-temperature saturation, the gas is cooled down with a gas cooler (M&C TechGroup Germany, model: ECP1000C) to a defined setpoint (2 °C – 15 °C), leading to a well-defined- saturation with subsequent separation of excess water vapor by condensation. Downstream of the gas cooler, a second condensation trap is positioned as a fail-safe mechanism for the gas cooler to prevent condensation from entering the piping system. The saturated gas is then utilized to operate low-delta-pressure mass flow controllers (Bronkhorst Deutschland Nord GmbH) at an operational pressure of 1.3 bar(a), which feeds a humidified partial gas flow into the dynamic dilution system. The inlet pressure of the mass flow controllers is precisely controlled with an upstream pressure regulator (Bronkhorst Deutschland Nord GmbH, EL-PRESS P-702CV), which simultaneously regulates the saturation pressure. Additionally, the pressure in the washing flask is continuously monitored by a pressure sensor (KELLER Druckmesstechnik AG, model: PAA-23SX) positioned upstream of the washing flask. The deviation between the regulated pressure and the measured pressure is at a scale of 0.001 bar(a), ensuring highly stable and accurate pressure control within the humidification system.

Consequently, and for example, when the HU operates at a total input flow rate of 300 $mL_n$/min while the mass flow controller in the HU regulates an output flow of 10 $mL_n$/min, an excess of 290 $mL_n$/min is discharged downstream of the pressure regulator at atmospheric conditions. This excess of humidified gas is continuously analyzed using a reference dew point mirror (MBW, model: 473-SHX), enabling real-time, *in situ*, and *in operando* quantification of the saturation volume fractions used for dynamic dilution.

## 2. Ammonia absorption band

Figure S2 demonstrates that the spectral window used for ammonia quantification is free from water absorption bands in the investigated measurement range. The water absorption band shown in Figure ***S2*** d) becomes observable only at humidity levels of several hundred $ppm_V$. Additionally, this water absorption band is implemented in AP2E's constrained fitting algorithm, which uses a reduced number of degrees of freedom to accurately determine the integrated ammonia absorbance.

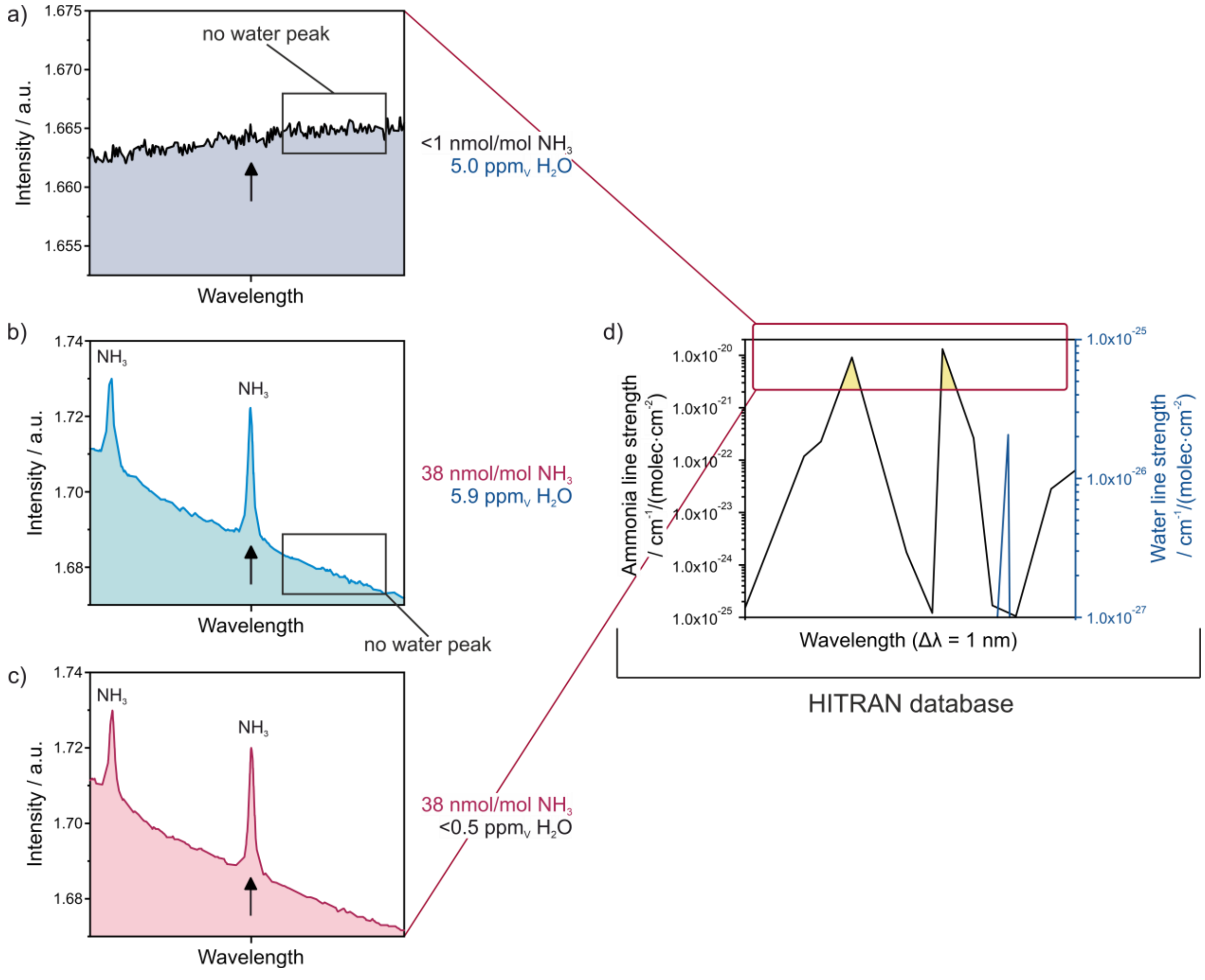


**Figure S2**: Ammonia absorption spectra demonstrating the absence of cross-sensitivity from trace humidity within the analyzed spectral window and measurement range. (a) Spectrum of 5 $ppm_V$ trace humidity in clean hydrogen with an amount of ammonia below the limit of quantification (<1 nmol/mol). (b) Spectrum of an amount of 38 nmol/mol ammonia with 5.9 $ppm_V$ trace humidity. (c) Spectrum of clean and dry hydrogen containing an amount of 38 nmol/mol ammonia. (d) Absorption spectrum of the analyzed spectral region ($\Delta\lambda$ = 1 nm) based on the HITRAN database[1]. The line strength shows that ammonia quantification within this range is highly selective and, more importantly, unaffected by trace humidity, with a line strength difference of $10^6$ $cm^{-1}/(molec \cdot cm^{-2})$ between ammonia and water.

## 3. Gas cell mirrors

Figure S3 shows two of the three mirrors mounted into the gas cell of the OF-CEAS system used. The diameter of each mirror is approximately 0.7 cm.

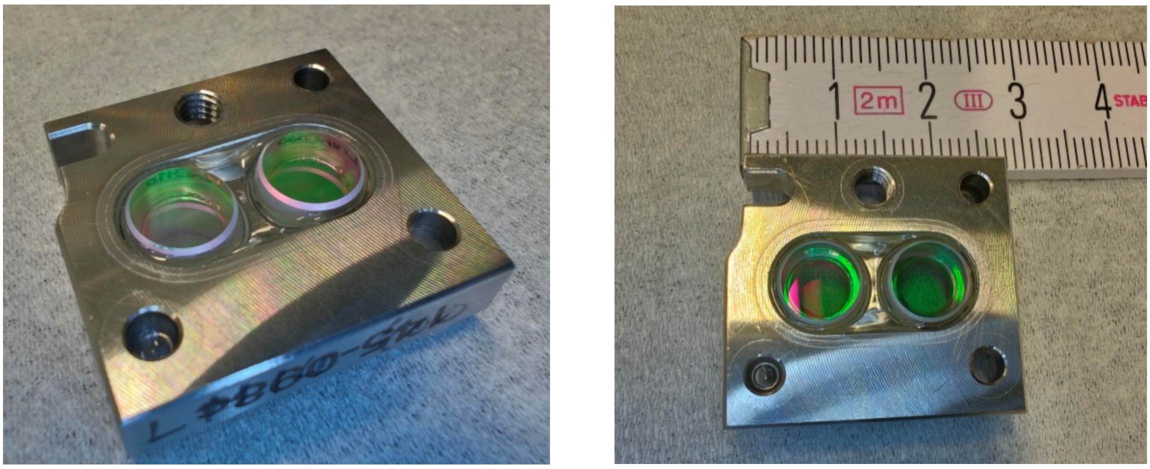

**Figure S3**: Image of the mirrors mounted in the ammonia gas cell with a V-shaped cavity.

## 4. Adsorption and desorption rates

Figure S4 presents an example of linear regression used to determine the rates from a trace-humidified to a dry state (Figure S4a) and from a dry to a trace-humidified state (Figure S4b).

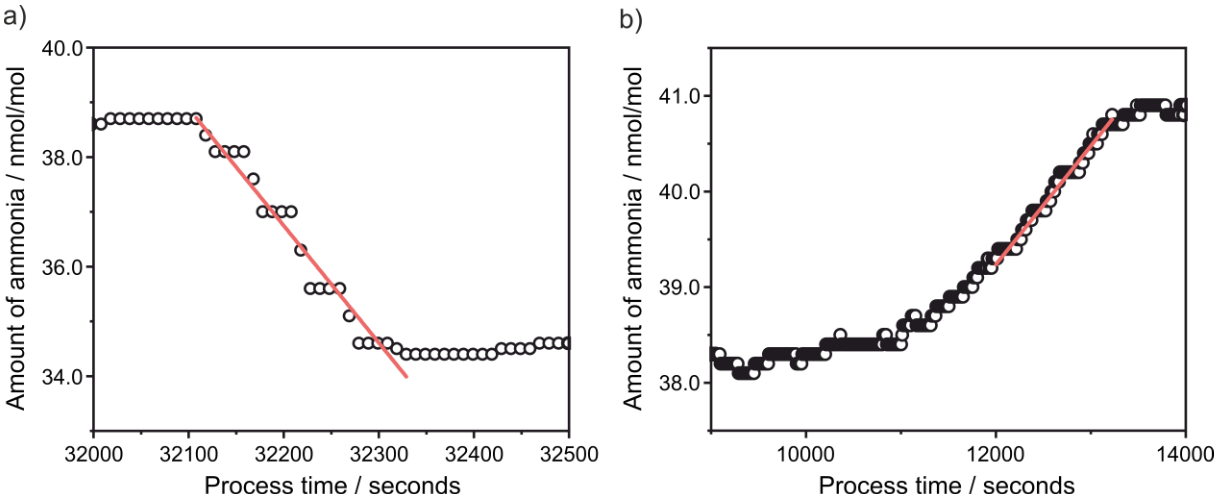


**Figure S4**: (a) Linear regression of the transition from a trace-humidified state to a dry state, and (b) linear regression for the transition from a dry state to a trace-humidified phase.

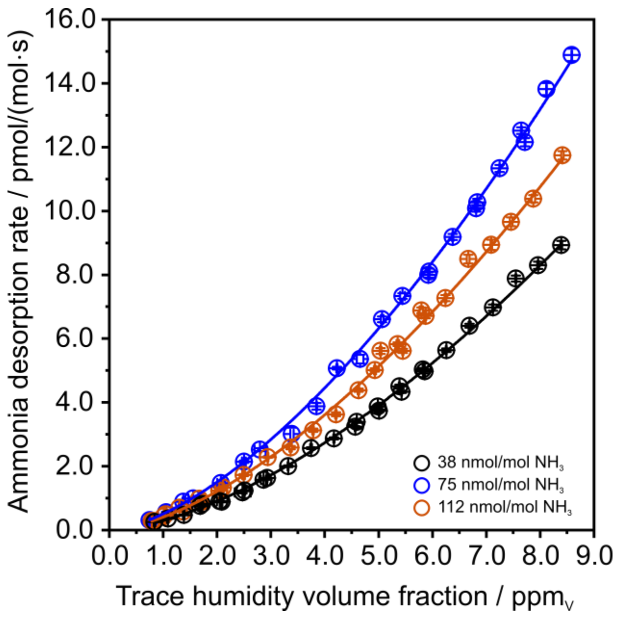


**Figure S5**: Ammonia desorption rates observed in the transition from dry to humidified hydrogen.

**Table S1**: An overview of all determined rates, investigated trace-humidity volume fractions (derived from frost point temperatures and line pressure), amounts of ammonia as well as important environmental parameters (temperature & pressure) for the measurements conducted over a measurement period of 4 months.

| Start of measurement (yyyy-mm-dd) | Measurement series* (chronological) | $x_{\mathrm{NH_3}}$ / µmol/mol | $SD(x_{\mathrm{NH_3}})$ / µmol/mol | $t_{\mathrm{f}}$/ °C | $SD(t_{\mathrm{f}})$ / K | $p_{\mathrm{L}}$ / Pa | $SD(p_{\mathrm{L}})$ / Pa | $\Phi_{\mathrm{w}}$ / ppm$_{\mathrm{V}}$ | $SD(\Phi_{\mathrm{w}})$ / ppm$_{\mathrm{V}}$ | $t_{\mathrm{a}}$/ °C | $SD(t_{\mathrm{a}})$ / K | $r$ (humid → dry) / µmol/(mol·s) | $U(r)$ (humid → dry) / µmol/(mol·s) | $r$ (dry → humid) / µmol/(mol·s) | $U(r)$ (dry → humid) / µmol/(mol·s) |
|---|---|---|---|---|---|---|---|---|---|---|---|---|---|---|---|
| 2024-09-28 | 4 | 0.03901 | 8.14E-05 | -77.299 | 0.060 | 101720.1 | 39.2 | 0.839 | 0.016 | 23.032 | 0.067 | -8.794E-06 | 9.639E-07 | 2.587E-07 | 5.342E-09 |
| 2024-09-29 | 4 | 0.03880 | 6.01E-05 | -75.633 | 0.042 | 102346.7 | 13.6 | 1.087 | 0.014 | 23.009 | 0.065 | -1.182E-05 | 1.469E-06 | 3.603E-07 | 2.107E-08 |
| 2024-09-29 | 4 | 0.03885 | 1.09E-04 | -74.086 | 0.030 | 102353.9 | 8.9 | 1.383 | 0.013 | 23.060 | 0.062 | -1.460E-05 | 1.572E-06 | 4.751E-07 | 1.679E-08 |
| 2024-09-30 | 4 | 0.03850 | 4.66E-05 | -72.667 | 0.030 | 101952.9 | 18.5 | 1.727 | 0.016 | 23.061 | 0.069 | -1.824E-05 | 1.728E-06 | 8.162E-07 | 1.758E-08 |
| 2024-09-30 | 4 | 0.03855 | 1.21E-04 | -71.446 | 0.015 | 101378.6 | 26.6 | 2.090 | 0.011 | 23.027 | 0.099 | -2.257E-05 | 3.198E-06 | 8.847E-07 | 3.156E-08 |
| 2024-10-01 | 4 | 0.03826 | 1.04E-04 | -70.269 | 0.019 | 100707.3 | 8.8 | 2.510 | 0.014 | 23.128 | 0.054 | -2.139E-05 | 1.750E-06 | 1.239E-06 | 3.498E-08 |
| 2024-10-01 | 4 | 0.03826 | 1.04E-04 | -69.243 | 0.010 | 100472.9 | 13.9 | 2.930 | 0.009 | 23.005 | 0.016 | -2.657E-05 | 3.654E-06 | 1.632E-06 | 2.121E-08 |
| 2024-10-02 | 4 | 0.03750 | 8.20E-05 | -68.396 | 0.008 | 100432.0 | 22.8 | 3.320 | 0.008 | 22.817 | 0.057 | -2.949E-05 | 2.666E-06 | 1.999E-06 | 5.669E-08 |
| 2024-10-02 | 4 | 0.03790 | 7.42E-05 | -67.548 | 0.015 | 100730.1 | 18.6 | 3.746 | 0.015 | 22.924 | 0.057 | -3.375E-05 | 3.073E-06 | 2.566E-06 | 6.113E-08 |
| 2024-10-03 | 4 | 0.03781 | 4.84E-05 | -66.796 | 0.010 | 100925.3 | 17.3 | 4.169 | 0.011 | 22.887 | 0.032 | -3.697E-05 | 4.261E-06 | 2.873E-06 | 7.093E-08 |
| 2024-10-03 | 4 | 0.03779 | 4.63E-05 | -66.100 | 0.004 | 101216.0 | 18.8 | 4.595 | 0.005 | 22.919 | 0.018 | -3.962E-05 | 5.655E-06 | 3.389E-06 | 6.372E-08 |
| 2024-10-04 | 4 | 0.03776 | 5.52E-05 | -65.487 | 0.012 | 101376.2 | 17.3 | 5.007 | 0.017 | 22.877 | 0.085 | -4.346E-05 | 5.387E-06 | 3.738E-06 | 9.970E-08 |
| 2024-10-04 | 4 | 0.03796 | 5.80E-05 | -64.911 | 0.004 | 101491.7 | 14.0 | 5.428 | 0.006 | 22.887 | 0.024 | -4.052E-05 | 2.966E-06 | 4.332E-06 | 7.706E-08 |
| 2024-10-05 | 4 | 0.03807 | 4.80E-05 | -64.370 | 0.004 | 101525.4 | 7.9 | 5.857 | 0.007 | 22.855 | 0.016 | -3.953E-05 | -7.906E-05 | 4.977E-06 | 1.287E-07 |
| 2024-10-12 | 5 | 0.03862 | 8.04E-05 | -72.919 | 0.052 | 100444.7 | 59.1 | 1.687 | 0.027 | 22.837 | 0.021 | -1.801E-05 | 2.174E-06 | 7.506E-07 | 2.131E-08 |
| 2024-10-13 | 5 | 0.03796 | 5.77E-05 | -71.672 | 0.080 | 100082.0 | 28.2 | 2.046 | 0.050 | 22.883 | 0.023 | -2.136E-05 | 3.988E-06 | 9.048E-07 | 2.577E-08 |
| 2024-10-13 | 5 | 0.03823 | 6.70E-5 | -70.35 | 0.239 | 100995.4 | 27.1 | 2.476 | 0.147 | 22.815 | 0.024 | -2.181E-05 | 2.165E-06 | 1.176E-06 | 3.252E-04 |
| 2024-10-14 | 5 | 0.03839 | 5.53E-05 | -69.345 | 0.058 | 101317.0 | 8.3 | 2.863 | 0.049 | 22.781 | 0.054 | -2.615E-05 | 3.148E-06 | 1.578E-06 | 4.360E-08 |
| 2024-10-14 | 5 | 0.03852 | 6.51E-05 | -66.128 | 0.008 | 101451.2 | 23.0 | 4.566 | 0.009 | 22.860 | 0.022 | -4.014E-05 | 6.710E-06 | 3.233E-06 | 5.337E-08 |
| 2024-10-15 | 5 | 0.03841 | 1.12E-04 | -65.471 | 0.006 | 102005.1 | 11.0 | 4.988 | 0.008 | 22.826 | 0.021 | -4.371E-05 | 6.314E-06 | 3.875E-06 | 7.963E-08 |
| 2024-10-15 | 5 | 0.03841 | 1.12E-04 | -64.918 | 0.005 | 102153.8 | 25.5 | 5.387 | 0.008 | 22.610 | 0.031 | -3.938E-05 | 3.685E-06 | 4.499E-06 | 1.268E-07 |
| 2024-10-16 | 5 | 0.03836 | 1.05E-04 | -64.381 | 0.005 | 101950.6 | 16.8 | 5.824 | 0.008 | 22.637 | 0.021 | -4.146E-05 | 4.452E-06 | 5.032E-06 | 9.975E-08 |
| 2024-10-16 | 5 | 0.03835 | 8.32E-05 | -63.902 | 0.005 | 101502.4 | 20.8 | 6.256 | 0.009 | 22.717 | 0.021 | -4.541E-05 | 4.200E-06 | 5.634E-06 | 1.145E-07 |
| 2024-10-17 | 5 | 0.03781 | 5.35E-05 | -63.437 | 0.008 | 101290.0 | 24.2 | 6.691 | 0.018 | 22.776 | 0.036 | -4.692E-05 | 5.153E-06 | 6.403E-06 | 1.190E-07 |
| 2024-10-17 | 5 | 0.03802 | 6.15E-05 | -62.990 | 0.004 | 101277.1 | 8.1 | 7.123 | 0.008 | 22.849 | 0.018 | -5.145E-05 | 8.439E-06 | 6.973E-06 | 2.036E-07 |

| | | | | | | | | | | | | | | |
|---|---|---|---|---|---|---|---|---|---|---|---|---|---|---|
| 2024-10-18 | 5 | 0.03781 | 8.06E-05 | -62.574 | 0.006 | 101253.9 | 18.8 | 7.547 | 0.012 | 22.939 | 0.030 | -5.291E-05 | 4.748E-06 | 7.881E-06 | 1.911E-07 |
| 2024-10-18 | 5 | 0.03774 | 8.72E-05 | -62.202 | 0.008 | 101050.9 | 17.8 | 7.961 | 0.019 | 22.969 | 0.029 | -5.829E-05 | 7.154E-06 | 8.296E-06 | 2.220E-07 |
| 2024-10-19 | 5 | 0.03773 | 6.67E-05 | -61.802 | 0.008 | 101295.8 | 18.8 | 8.393 | 0.018 | 23.009 | 0.046 | -6.054E-05 | 7.560E-06 | 8.933E-06 | 2.610E-07 |
| 2024-08-31 | 1 | 0.07465 | 1.00E-04 | -65.354 | 0.037 | 102128.1 | 7.9 | 5.066 | 0.026 | 23.114 | 0.033 | -5.851E-05 | 6.712E-06 | 6.605E-06 | 1.772E-07 |
| 2024-08-31 | 1 | 0.07438 | 9.96E-05 | -64.255 | 0.009 | 102008.4 | 18.9 | 5.924 | 0.007 | 22.890 | 0.021 | -6.794E-05 | 1.047E-05 | 7.993E-06 | 1.675E-07 |
| 2024-09-01 | 1 | 0.07507 | 1.36E-04 | -63.275 | 0.007 | 101819.8 | 20.1 | 6.809 | 0.007 | 23.175 | 0.062 | -7.717E-05 | 7.329E-06 | 1.008E-05 | 2.058E-07 |
| 2024-09-01 | 1 | 0.07455 | 8.71E-05 | -62.466 | 0.007 | 101392.2 | 8.1 | 7.651 | 0.007 | 22.924 | 0.029 | -8.894E-05 | 1.157E-05 | 1.251E-05 | 2.104E-07 |
| 2024-09-04 | 2 | 0.07514 | 7.38E-05 | -66.695 | 0.017 | 101057.7 | 16.1 | 4.225 | 0.010 | 23.030 | 0.052 | -5.064E-05 | 5.955E-06 | 5.074E-06 | 8.851E-08 |
| 2024-09-05 | 2 | 0.07449 | 1.03E-04 | -64.921 | 0.060 | 101052.4 | 19.0 | 5.444 | 0.046 | 22.585 | 0.014 | -6.442E-05 | 5.855E-06 | 7.336E-06 | 1.687E-07 |
| 2024-09-06 | 2 | 0.07526 | 6.89E-05 | -64.295 | 0.007 | 101149.4 | 8.2 | 5.941 | 0.006 | 22.891 | 0.020 | -6.792E-05 | 7.107E-06 | 8.100E-06 | 1.911E-07 |
| 2024-09-06 | 2 | 0.07439 | 7.74E-05 | -63.803 | 0.012 | 100998.3 | 15.9 | 6.376 | 0.010 | 22.647 | 0.034 | -7.331E-05 | 8.973E-06 | 9.187E-06 | 1.923E-07 |
| 2024-09-07 | 2 | 0.07439 | 7.74E-05 | -63.299 | 0.005 | 101088.0 | 20.0 | 6.835 | 0.005 | 23.106 | 0.033 | -7.945E-05 | 1.011E-05 | 1.027E-05 | 2.235E-07 |
| 2024-09-07 | 2 | 0.07440 | 4.99E-05 | -62.894 | 0.007 | 100860.0 | 8.2 | 7.247 | 0.008 | 22.645 | 0.019 | -8.275E-05 | 8.846E-06 | 1.133E-05 | 2.241E-07 |
| 2024-09-08 | 2 | 0.07478 | 6.57E-05 | -62.458 | 0.006 | 100620.9 | 9.4 | 7.717 | 0.007 | 22.975 | 0.050 | -8.139E-05 | 8.002E-06 | 1.215E-05 | 2.486E-07 |
| 2024-09-08 | 2 | 0.07445 | 1.18E-04 | -62.127 | 0.006 | 100110.5 | 38.3 | 8.119 | 0.006 | 22.756 | 0.017 | -9.855E-05 | 1.122E-05 | 1.382E-05 | 3.778E-07 |
| 2024-09-09 | 2 | 0.07459 | 5.71E-05 | -61.764 | 0.006 | 99515.1 | 20.6 | 8.587 | 0.007 | 22.972 | 0.033 | -1.005E-04 | 9.626E-06 | 1.489E-05 | 3.940E-07 |
| 2024-09-12 | 3 | 0.07501 | 1.20E-04 | -78.069 | 0.175 | 100826.2 | 12.2 | 0.749 | 0.021 | 22.749 | 0.035 | -1.110E-05 | 1.690E-06 | 3.149E-07 | 9.755E-09 |
| 2024-09-12 | 3 | 0.07528 | 8.79E-05 | -75.887 | 0.026 | 101063.0 | 7.4 | 1.057 | 0.004 | 22.615 | 0.029 | -1.561E-05 | 1.525E-06 | 5.544E-07 | 3.439E-08 |
| 2024-09-13 | 3 | 0.07541 | 6.79E-05 | -74.197 | 0.068 | 101335.1 | 7.9 | 1.373 | 0.014 | 22.858 | 0.058 | -2.081E-05 | 2.383E-06 | 8.878E-07 | 2.737E-08 |
| 2024-09-13 | 3 | 0.07521 | 1.05E-04 | -73.377 | 0.355 | 101578.6 | 6.6 | 1.560 | 0.092 | 22.697 | 0.067 | -2.270E-05 | 2.116E-06 | 9.894E-07 | 5.268E-08 |
| 2024-09-14 | 3 | 0.07512 | 1.07E-04 | -71.481 | 0.219 | 101799.4 | 18.5 | 2.073 | 0.066 | 22.795 | 0.062 | -3.144E-05 | 4.312E-06 | 1.470E-06 | 8.344E-08 |
| 2024-09-14 | 3 | 0.07512 | 1.07E-04 | -70.223 | 0.101 | 101875.0 | 19.4 | 2.500 | 0.037 | 22.630 | 0.072 | -3.787E-05 | 4.539E-06 | 2.141E-06 | 7.917E-08 |
| 2024-09-15 | 3 | 0.07509 | 1.26E-04 | -69.488 | 0.253 | 101577.1 | 10.0 | 2.798 | 0.102 | 22.722 | 0.081 | -4.126E-05 | 3.510E-06 | 2.521E-06 | 8.685E-08 |
| 2024-09-15 | 3 | 0.07495 | 1.19E-04 | -68.184 | 0.059 | 101471.0 | 25.1 | 3.391 | 0.029 | 22.953 | 0.067 | -3.909E-05 | 3.061E-06 | 3.013E-06 | 4.009E-07 |
| 2024-09-16 | 3 | 0.07490 | 1.43E-04 | -67.308 | 0.016 | 101516.6 | 19.1 | 3.849 | 0.008 | 23.205 | 0.015 | -4.536E-05 | 3.940E-06 | 3.881E-06 | 2.131E-07 |
| 2024-09-16 | 3 | 0.07461 | 7.74E-05 | -65.959 | 0.098 | 101912.6 | 13.0 | 4.658 | 0.062 | 23.079 | 0.019 | -5.454E-05 | 5.296E-06 | 5.359E-06 | 2.743E-07 |
| 2024-10-25 | 6 | 0.1119 | 1.10E-04 | -77.429 | 0.046 | 101577.3 | 15.6 | 0.823 | 0.012 | 22.900 | 0.021 | -1.206E-05 | 2.129E-06 | 2.791E-07 | 2.010E-08 |
| 2024-10-26 | 6 | 0.1119 | 8.78E-05 | -75.911 | 0.056 | 101687.3 | 9.0 | 1.047 | 0.019 | 22.679 | 0.027 | -1.466E-05 | 1.929E-06 | 4.949E-07 | 1.688E-08 |
| 2024-10-26 | 6 | 0.1122 | 2.04E-04 | -74.181 | 0.033 | 101463.5 | 20.5 | 1.375 | 0.015 | 22.981 | 0.020 | -1.981E-05 | 2.555E-06 | 5.784E-07 | 3.291E-08 |
| 2024-10-27 | 6 | 0.1116 | 1.18E-04 | -72.811 | 0.042 | 101265.4 | 17.4 | 1.701 | 0.022 | 22.763 | 0.026 | -2.544E-05 | 3.817E-06 | 8.765E-07 | 1.846E-08 |

| | | | | | | | | | | | | | | | |
|---|---|---|---|---|---|---|---|---|---|---|---|---|---|---|---|
| 2024-10-28 | 6 | 0.1121 | 1.18E-04 | -71.347 | 0.023 | 101922.0 | 39.8 | 2.111 | 0.014 | 22.915 | 0.020 | -3.208E-05 | 4.155E-06 | 1.332E-06 | 8.805E-08 |
| 2024-10-28 | 6 | 0.1118 | 8.32E-05 | -70.230 | 0.033 | 101938.9 | 13.5 | 2.495 | 0.025 | 22.699 | 0.023 | -3.650E-05 | 4.564E-06 | 1.724E-06 | 6.512E-08 |
| 2024-10-29 | 6 | 0.1119 | 7.66E-05 | -68.200 | 0.025 | 101952.0 | 22.2 | 3.366 | 0.024 | 22.963 | 0.052 | -3.944E-05 | 3.601E-06 | 2.583E-06 | 1.021E-07 |
| 2024-10-29 | 6 | 0.1121 | 1.52E-04 | -67.403 | 0.010 | 101889.2 | 9.5 | 3.783 | 0.012 | 23.028 | 0.017 | -4.605E-05 | 4.773E-06 | 3.127E-06 | 1.028E-07 |
| 2024-10-30 | 6 | 0.1126 | 9.91E-05 | -66.658 | 0.012 | 101995.1 | 19.8 | 4.208 | 0.014 | 23.074 | 0.022 | -4.974E-05 | 4.634E-06 | 3.626E-06 | 1.215E-07 |
| 2024-10-30 | 6 | 0.1129 | 8.26E-05 | -65.987 | 0.011 | 102156.1 | 14.3 | 4.627 | 0.015 | 23.033 | 0.018 | -5.447E-05 | 5.356E-06 | 4.384E-06 | 9.723E-08 |
| 2024-10-31 | 6 | 0.1120 | 6.97E-05 | -65.385 | 0.012 | 102274.4 | 15.8 | 5.036 | 0.017 | 22.895 | 0.026 | -5.812E-05 | 6.303E-06 | 5.615E-06 | 1.826E-07 |
| 2024-10-31 | 6 | 0.1122 | 1.03E-04 | -64.856 | 0.008 | 101879.7 | 30.5 | 5.449 | 0.013 | 22.939 | 0.017 | -6.455E-05 | 1.178E-05 | 5.618E-06 | 1.328E-07 |
| 2024-11-01 | 6 | 0.1122 | 1.07E-04 | -64.346 | 0.009 | 101609.3 | 8.4 | 5.872 | 0.014 | 22.829 | 0.018 | -6.682E-05 | 9.995E-06 | 6.711E-06 | 1.415E-07 |
| 2024-11-02 | 7 | 0.1131 | 9.74E-05 | -76.065 | 0.039 | 102769.9 | 25.0 | 1.011 | 0.012 | 22.462 | 0.027 | -1.445E-05 | 2.173E-06 | 4.292E-07 | 1.619E-08 |
| 2024-11-02 | 7 | 0.1131 | 6.45E-05 | -74.464 | 0.033 | 102736.3 | 17.1 | 1.300 | 0.014 | 22.250 | 0.023 | -2.022E-05 | 2.894E-06 | 6.959E-07 | 3.029E-08 |
| 2024-11-03 | 7 | 0.1133 | 1.29E-04 | -72.872 | 0.028 | 102335.3 | 9.2 | 1.668 | 0.014 | 22.436 | 0.026 | -2.357E-05 | 3.991E-06 | 9.779E-07 | 3.040E-08 |
| 2024-11-10 | 8 | 0.1130 | 1.34E-04 | -71.533 | 0.025 | 102424.8 | 11.6 | 2.042 | 0.015 | 22.617 | 0.102 | -2.912E-05 | 4.717E-06 | 1.240E-06 | 7.671E-08 |
| 2024-11-10 | 8 | 0.1128 | 1.14E-04 | -70.388 | 0.015 | 102485.8 | 16.3 | 2.424 | 0.011 | 22.564 | 0.099 | -3.575E-05 | 3.446E-06 | —** | —** |
| 2024-11-11 | 8 | 0.1128 | 1.21E-04 | -69.328 | 0.019 | 102325.1 | 16.9 | 2.842 | 0.016 | 22.543 | 0.088 | -4.232E-05 | 6.538E-06 | 2.108E-06 | 1.911E-07 |
| 2024-11-11 | 8 | 0.1126 | 1.07E-04 | -65.531 | 0.014 | 102301.0 | 17.5 | 4.931 | 0.019 | 22.560 | 0.068 | -5.863E-05 | 8.867E-06 | 5.017E-06 | 1.045E-07 |
| 2024-11-12 | 8 | 0.1124 | 1.23E-04 | -64.916 | 0.016 | 102777.3 | 16.2 | 5.356 | 0.024 | 22.512 | 0.087 | -6.536E-05 | 6.453E-06 | 5.824E-06 | 1.145E-07 |
| 2024-11-12 | 8 | 0.1123 | 9.45E-05 | -64.362 | 0.019 | 102759.6 | 20.4 | 5.794 | 0.031 | 22.465 | 0.083 | -6.984E-05 | 1.121E-05 | 6.885E-06 | 1.846E-07 |
| 2024-11-13 | 8 | 0.1123 | 9.45E-05 | -63.856 | 0.011 | 102403.9 | 16.0 | 6.242 | 0.019 | 22.520 | 0.057 | -7.269E-05 | 1.170E-05 | 7.267E-06 | 1.862E-07 |
| 2024-11-13 | 8 | 0.1123 | 9.02E-05 | -63.401 | 0.013 | 102213.7 | 9.1 | 6.665 | 0.024 | 22.629 | 0.077 | -7.736E-05 | 1.270E-05 | 8.494E-06 | 2.753E-07 |
| 2024-11-14 | 8 | 0.1126 | 1.13E-04 | -62.996 | 0.007 | 101626.4 | 17.0 | 7.092 | 0.015 | 22.578 | 0.077 | -8.537E-05 | 1.039E-05 | 8.936E-06 | 3.190E-07 |
| 2024-12-28 | 9 | 0.1118 | 3.69E-04 | -62.469 | 0.011 | 102895.3 | 13.6 | 7.536 | 0.022 | 23.426 | 0.015 | -9.291E-05 | 7.278E-06 | —** | —** |
| 2024-12-28 | 9 | 0.1120 | 4.12E-04 | -62.091 | 0.008 | 102642.6 | 7.7 | 7.960 | 0.018 | 23.357 | 0.012 | -9.534E-05 | 8.803E-06 | —** | —** |
| 2024-12-27 | 9 | 0.1120 | 4.12E-04 | -61.705 | 0.009 | 102412.5 | 18.3 | 8.413 | 0.022 | 23.356 | 0.026 | -1.022E-04 | 7.784E-06 | 1.174E-05 | 2.635E-07 |

* In this context, “measurement series” means that the measurement cycles were performed continuously in a consecutive way.

**Measurement points affected by external interferences – such as strong fluctuations at ambient temperature – that caused signal drifts were excluded from the data analysis.

A 2-hour time window (720 data points, recorded at intervals of 10 seconds) was used to calculate the corresponding values listed for $x_{\mathrm{NH_3}}$ (pre-phase data), $t_\mathrm{f}$, and $\Phi_\mathrm{w}$. This time window of 2 hours was positioned immediately ahead of the respective transition phases. For $p_\mathrm{L}$ and $t_\mathrm{a}$, a 2-hour time window within the humidification phase was used (also prior to the transition).

## 5. Bias

The bias was calculated by the following equation:

$$Bias = \frac{\left(x_{NH_3}(\text{humid}) - x_{NH_3}(\text{dry pre-phase})\right) + \left(x_{NH_3}(\text{humid}) - x_{NH_3}(\text{dry post-phase})\right)}{2}$$

**Table S2**: An overview of the amounts of ammonia in the humidified state, pre-, and post-phase that were used to calculate the bias in trace-humidified state.

| $x_{NH_3}$ in the pre-phase / nmol/mol | $x_{NH_3}$ in the post-phase / nmol/mol | $x_{NH_3}$ in the trace-humidified phase / nmol/mol | *Bias* / nmol/mol | *U*(*Bias*) / nmol/mol | $\Phi_w$ / ppm$_V$ | *SD*($\Phi_w$) / ppm$_V$ |
|---|---|---|---|---|---|---|
| 39.0 | 39.0 | 39.4 | 0.40 | 0.12 | 0.839 | 0.016 |
| 38.7 | 38.8 | 39.2 | 0.45 | 0.12 | 1.087 | 0.014 |
| 38.8 | 38.5 | 39.2 | 0.55 | 0.12 | 1.383 | 0.013 |
| 38.5 | 38.5 | 39.1 | 0.60 | 0.12 | 1.727 | 0.016 |
| 38.5 | 38.2 | 39.0 | 0.65 | 0.12 | 2.090 | 0.011 |
| 38.1 | 37.8 | 38.8 | 0.85 | 0.12 | 2.510 | 0.014 |
| 37.4 | 37.6 | 38.5 | 1.00 | 0.12 | 3.320 | 0.008 |
| 37.9 | 37.9 | 38.6 | 0.70 | 0.12 | 3.746 | 0.015 |
| 37.8 | 37.8 | 38.7 | 0.90 | 0.12 | 4.169 | 0.011 |
| 37.8 | 37.8 | 38.8 | 1.00 | 0.12 | 4.595 | 0.005 |
| 37.7 | 37.9 | 38.7 | 0.90 | 0.12 | 5.007 | 0.017 |
| 37.9 | 38.0 | 38.8 | 0.85 | 0.12 | 5.428 | 0.006 |
| 38.0 | 38.0 | 39.0 | 1.00 | 0.12 | 5.857 | 0.007 |
| 38.6 | 37.9 | 38.8 | 0.55 | 0.12 | 1.687 | 0.027 |
| 38.0 | 38.2 | 38.8 | 0.70 | 0.12 | 2.046 | 0.050 |
| 38.2 | 38.4 | 38.9 | 0.60 | 0.12 | 2.476 | 0.147 |
| 38.4 | 38.5 | 39.1 | 0.65 | 0.12 | 2.863 | 0.049 |
| 38.5 | 38.3 | 39.2 | 0.80 | 0.12 | 4.566 | 0.009 |
| 38.3 | 38.7 | 39.3 | 0.80 | 0.12 | 4.988 | 0.008 |
| 38.7 | 38.4 | 39.2 | 0.65 | 0.12 | 5.387 | 0.008 |
| 38.3 | 38.3 | 39.2 | 0.90 | 0.12 | 5.824 | 0.008 |
| 38.3 | 37.9 | 38.9 | 0.80 | 0.12 | 6.256 | 0.009 |
| 37.8 | 38.0 | 39.0 | 1.10 | 0.12 | 6.691 | 0.018 |
| 38.0 | 37.8 | 38.7 | 0.80 | 0.12 | 7.123 | 0.008 |
| 37.9 | 37.7 | 38.6 | 0.80 | 0.12 | 7.547 | 0.012 |
| 37.6 | 37.7 | 38.7 | 1.05 | 0.12 | 7.961 | 0.019 |
| 37.7 | 37.7 | 38.6 | 0.90 | 0.12 | 8.393 | 0.018 |
| 74.7 | 74.4 | 75.3 | 0.75 | 0.12 | 5.066 | 0.026 |
| 74.3 | 75.2 | 75.5 | 0.75 | 0.12 | 5.924 | 0.007 |
| 75.0 | 74.6 | 75.8 | 1.00 | 0.12 | 6.809 | 0.007 |
| 74.6 | 75.0 | 75.8 | 1.00 | 0.12 | 7.651 | 0.007 |
| 75.2 | 74.0 | 75.2 | 0.64 | 0.12 | 4.225 | 0.010 |
| 75.4 | 74.4 | 75.9 | 1.00 | 0.12 | 5.941 | 0.006 |
| 74.4 | 74.6 | 75.6 | 1.10 | 0.12 | 6.376 | 0.010 |
| 75.3 | 75.3 | 76.5 | 1.20 | 0.12 | 6.835 | 0.005 |
| 74.4 | 74.3 | 75.4 | 1.05 | 0.12 | 7.247 | 0.008 |

| | | | | | | |
|---|---|---|---|---|---|---|
| 74.6 | 74.5 | 75.5 | 0.95 | 0.12 | 7.717 | 0.007 |
| 74.5 | 74.4 | 75.4 | 0.95 | 0.12 | 8.119 | 0.006 |
| 74.6 | 74.9 | 75.9 | 1.15 | 0.12 | 8.587 | 0.007 |
| 75.3 | 75.3 | 75.6 | 0.30 | 0.12 | 1.057 | 0.004 |
| 75.4 | 75.2 | 75.8 | 0.50 | 0.12 | 1.373 | 0.014 |
| 75.2 | 75.1 | 75.5 | 0.35 | 0.12 | 1.560 | 0.092 |
| 75.1 | 75.2 | 75.8 | 0.65 | 0.12 | 2.073 | 0.066 |
| 75.2 | 75.0 | 75.8 | 0.70 | 0.12 | 2.500 | 0.037 |
| 75.0 | 75.0 | 75.8 | 0.80 | 0.12 | 2.798 | 0.102 |
| 74.9 | 74.7 | 75.5 | 0.70 | 0.12 | 3.391 | 0.029 |
| 75.0 | 74.6 | 75.5 | 0.70 | 0.12 | 3.849 | 0.008 |
| 74.6 | 74.4 | 75.4 | 0.90 | 0.12 | 4.658 | 0.062 |
| 111.7 | 111.7 | 112.0 | 0.30 | 0.12 | 0.823 | 0.012 |
| 112.0 | 112.2 | 112.5 | 0.40 | 0.12 | 1.047 | 0.019 |
| 112.1 | 111.7 | 112.4 | 0.50 | 0.12 | 1.375 | 0.015 |
| 111.6 | 112.2 | 112.6 | 0.70 | 0.12 | 1.701 | 0.022 |
| 112.0 | 111.9 | 112.6 | 0.65 | 0.12 | 2.111 | 0.014 |
| 111.8 | 112.0 | 112.8 | 0.90 | 0.12 | 2.495 | 0.025 |
| 111.9 | 112.0 | 112.7 | 0.75 | 0.12 | 3.366 | 0.024 |
| 112.1 | 112.2 | 112.9 | 0.75 | 0.12 | 3.783 | 0.012 |
| 112.0 | 111.9 | 112.7 | 0.75 | 0.12 | 5.036 | 0.017 |
| 112.1 | 112.0 | 113.0 | 0.95 | 0.12 | 5.449 | 0.013 |
| 112.2 | 112.2 | 113.3 | 1.10 | 0.12 | 5.872 | 0.014 |
| 113.0 | 113.0 | 113.6 | 0.60 | 0.12 | 1.011 | 0.012 |
| 113.0 | 113.2 | 113.7 | 0.60 | 0.12 | 1.300 | 0.014 |
| 113.3 | 112.6 | 113.6 | 0.65 | 0.12 | 1.668 | 0.014 |
| 113.0 | 112.7 | 113.7 | 0.85 | 0.12 | 2.042 | 0.015 |
| 112.7 | 112.7 | 113.4 | 0.70 | 0.12 | 2.424 | 0.011 |
| 112.7 | 112.6 | 113.4 | 0.75 | 0.12 | 2.842 | 0.016 |
| 112.6 | 112.3 | 113.3 | 0.85 | 0.12 | 4.931 | 0.019 |
| 112.4 | 112.3 | 113.3 | 0.95 | 0.12 | 5.794 | 0.031 |
| 112.4 | 112.2 | 113.5 | 1.20 | 0.12 | 6.242 | 0.019 |
| 112.2 | 112.5 | 113.3 | 0.95 | 0.12 | 6.665 | 0.024 |
| 112.5 | 112.4 | 113.3 | 0.85 | 0.12 | 7.092 | 0.015 |
| 111.6 | 111.8 | 112.6 | 0.90 | 0.12 | 7.462 | 0.024 |
| 111.8 | 112.0 | 113.0 | 1.10 | 0.12 | 7.873 | 0.034 |
| 111.8 | 111.8 | 112.9 | 1.10 | 0.12 | 7.536 | 0.022 |
| 111.8 | 112.2 | 113.0 | 1.00 | 0.12 | 7.960 | 0.018 |
| 112.1 | 112.1 | 113.2 | 1.10 | 0.12 | 8.413 | 0.022 |

## Nomenclature

| | |
|---|---|
| $q_V$ | Flow rate |
| $SD$ | Standard deviation |
| $U$ | Uncertainty |
| $x_{\mathrm{NH_3}}$ | Amount of ammonia |
| $t_{\mathrm{f}}$ | Frost point temperature |
| $p_{\mathrm{L}}$ | Pipe pressure |
| $\Phi_{\mathrm{w}}$ | Trace-humidity volume fraction |
| $t_{\mathrm{a}}$ | Ambient temperature |
| $r$ | Reaction rate |

# References

(1) Gordon, I. E.; Rothman, L. S.; Hargreaves, R. J.; Hashemi, R.; Karlovets, E. V.; Skinner, F. M.; Conway, E. K.; Hill, C.; Kochanov, R. V.; Tan, Y.; Wcisło, P.; Finenko, A. A.; Nelson, K.; Bernath, P. F.; Birk, M.; Boudon, V.; Campargue, A.; Chance, K. V.; Coustenis, A.; Drouin, B. J.; Flaud, J.-M.; Gamache, R. R.; Hodges, J. T.; Jacquemart, D.; Mlawer, E. J.; Nikitin, A. V.; Perevalov, V. I.; Rotger, M.; Tennyson, J.; Toon, G. C.; Tran, H.; Tyuterev, V. G.; Adkins, E. M.; Baker, A.; Barbe, A.; Canè, E.; Császár, A. G.; Dudaryonok, A.; Egorov, O.; Fleisher, A. J.; Fleurbaey, H.; Foltynowicz, A.; Furtenbacher, T.; Harrison, J. J.; Hartmann, J.-M.; Horneman, V.-M.; Huang, X.; Karman, T.; Karns, J.; Kassi, S.; Kleiner, I.; Kofman, V.; Kwabia–Tchana, F.; Lavrentieva, N. N.; Lee, T. J.;Long, D. A.; Lukashevskaya, A. A.; Lyulin, O. M.; Makhnev, V. Yu.; Matt, W.; Massie, S. T.; Melosso, M.; Mikhailenko, S. N.; Mondelain, D.; Müller, H. S. P.; Naumenko, O. V.; Perrin, A.; Polyansky, O. L.; Raddaoui, E.; Raston, P. L.; Reed, Z. D.; Rey, M.; Richard, C.; Tóbiás, R.; Sadiek, I.; Schwenke, D. W.; Starikova, E.; Sung, K.; Tamassia, F.; Tashkun, S. A.; Vander Auwera, J.; Vasilenko, I. A.; Vigasin, A. A.; Villanueva, G. L.; Vispoel, B.; Wagner, G.; Yachmenev, A.; Yurchenko, S. N. The HITRAN2020 molecular spectroscopic database. *J. Quant. Spectrosc. Radiat. Transf.* **2022**, *277*, 107949. DOI: https://doi.org/10.1016/j.jqsrt.2021.107949.
